# Spectral Pattern of Chocolate Production: Early Detection of Quality Problems

**DOI:** 10.1111/1750-3841.71269

**Published:** 2026-07-20

**Authors:** Yağmur Küçükduman, İkra Doğa Korkmaz, Hüseyin Güray Çiftçi, Gökhan İpkin, Sinem Argün, Zeynep Pınar Kara, Gonca Bilge Özel, Özge Taştan Ülkü, Ömer Said Toker, Zeynep Mutlu

**Affiliations:** ^1^ Department of Food Engineering Faculty of Engineering and Natural Sciences Yeditepe University Istanbul Türkiye; ^2^ Department of Biotechnology, Graduate School Yeditepe University Istanbul Türkiye; ^3^ Department of Gastronomy and Culinary Arts, Faculty of Fine Arts Yeditepe University Istanbul Türkiye; ^4^ Department of Food Engineering, Faculty of Chemical and Metallurgical Engineering Yıldız Technical University Istanbul Türkiye; ^5^ Elvan Gıda San. ve Tic. A.Ş., Sefaköy Istanbul Türkiye

**Keywords:** chemometrics, chocolate, conching, cooling, FTIR, tempering

## Abstract

Quality defects in chocolate can be classified as physicochemical quality problems and structural instability (blooming). Both problems can be detected after production and generally take time to become visible. Therefore, early detection tools are necessary to prevent economic losses in the food industry. In this study, FTIR spectra collected at different stages of manufacturing were combined into spectral patterns to predict future quality defects, such as physicochemical defects (improper water activity, color, whiteness index, and hardness) and structural instability (fat and sugar bloom), using chemometrics. Accordingly, for uniform and defective chocolate production, five different conching processes, three different tempering processes, and two different cooling processes were used, and the samples were stored at 15°C, 20°C, and 28°C. To evaluate the defective samples, differential scanning calorimetry (DSC), polarized light microscopy, visual appearance tests for structural instability, and physicochemical analyses were conducted. Results showed that storage at 28°C caused fat bloom in all samples, regardless of processing. Therefore, to evaluate the effects of production parameters on fat bloom, 20°C samples were used in partial least squares‐discriminant analysis (PLS‐DA) models using spectral pattern. On the other hand, sugar bloom just appeared in the samples stored at 15°C, which were used for sugar bloom prediction PLS‐DA models. Although fat and sugar bloom required time (4–8 months), physicochemical defects (unsuitable water activity, hardness, and color) appeared even in new production. PLS‐DA models can predict these defects in new production, as well as formation of fat and sugar bloom during storage with 100% sensitivity and specificity in prediction.

**Practical Applications:**

The chocolate industry can use this FTIR‐based early prediction approach to reduce discarded products and economic losses and to improve the final product quality in the production line. This practical solution can be extended to different food matrices for monitoring of food production.

## 1 Introduction

Chocolate quality depends on processing and storage conditions, and it is sensitive to thermal and mechanical treatments (Caparosa and Hartel 2019; Toker et al. 2019). Key quality characteristics that adversely affect consumer acceptance and commercial viability include fat bloom, sugar bloom, and surface cracking (Bursa et al. 2023; Gutiérrez 2017). Chocolate bloom, which is a structural instability, causes a whitish surface layer that develops as a result of physicochemical changes in both the fat and sugar phases (Widlak and Hartel 2012). It is the general name for both sugar and fat bloom. Fat bloom develops via the migration of lipids and polymorphic transformations in cocoa butter crystals, mechanisms that are significantly affected by storage temperature, temperature fluctuation, and interaction among formulation ingredients (Clercq et al. 2014; Hodge and Rousseau 2002; Hřivna et al. 2021; Watanabe et al. 2023). Sugar bloom, on the other hand, occurs mainly due to moisture absorption, which causes sucrose to dissolve and then recrystallize on the chocolate's surface, particularly when exposed to elevated humidity or varying temperatures (Jawad et al. 2018; Son et al. 2018). Appropriate tempering techniques accelerate the formation of stable crystal structures in cocoa butter, thereby minimizing the risk of fat bloom during storage (Hodge and Rousseau 2002; Sonwai and Rousseau 2006). Furthermore, manufacturing processes, including conching and tempering, are critical for flavor enhancement and for maintaining long‐term structural integrity (Gutiérrez 2017; Machálková et al. 2015; Toker et al. 2019).

Techniques, such as confocal Raman microscopy, scanning electron microscopy (SEM), atomic force microscopy (AFM), polarized light microscopy, x‐ray techniques, confocal laser scanning, and profilometry, are used to observe chocolate blooming (Konar 2013). However, these classical methods have various drawbacks. For transmitted light microscopy, the solid chocolate sample must be melted and spread into a thin layer. Moreover, the sample needs to be diluted in fat, and the resolution of the microscope is limited (Feichtinger et al. 2020; Gaikwad 2012; Nijsse et al. 2024). The AFM technique, on the other hand, has some limitations due to the relatively small scanning area and low scanning speed of the microscope (Ricci and Braga 2004). This method is not suitable for industrial adaptation. Although SEM is used as a high‐resolution imaging device, its biggest disadvantages are the need for sample preparation and its inability to observe real‐time changes (Ashida et al. 2020). For differential scanning calorimetry (DSC), sample preparation is complex and costly (Gutiérrez 2017). Similarly, x‐ray methods are expensive and time‐consuming. To overcome these disadvantages, spectroscopic methods that do not require sample preparation and enable fast, online analysis can be used. They also allow multiple tests to be performed with a single sample (Nawrocka and Lamorska 2013).

Spectroscopic techniques can provide real‐time monitoring of food processes to improve the process economics and food quality. Although spectroscopic methods have been used in the food industry as bench‐top systems for more than 20 years, recently, with improvements in technology, these systems have begun to be produced as handheld or online systems. In particular, IR spectroscopy is widely used in the pharmaceutical industry (De Beer et al. 2011), dairy industry (Pu et al. 2020), and meat industry (Dixit et al. 2017) for process monitoring. Because these techniques provide chemical data on the food compound without using chemicals, they are rapid and essential for inline/online analysis. Recently, FTIR spectroscopy is no longer limited to bench‐top systems; portable and handheld systems have become very attractive to manufacturers. However, only a limited number of studies are available in the literature on the real‐time use of FTIR spectroscopy. In a study, dry film FTIR spectroscopy was used at line measurement for monitoring of enzymatic protein hydrolysate, which is produced with different process parameters to optimize the process (Kafle et al. 2024). In another study, FTIR spectroscopy with multivariate data analysis technique detected protein and carbohydrate differentiation successfully during fermentation process (Greulich et al. 2024). In the study conducted by Pavli et al. (2018), a relationship was established between microbial spoilage and FTIR spectra data with the aid of distinguishing quality classes of ham slices and predicting microbial counts. In a study reported by Cavaglia et al. (2019), ATR‐FTIR coupled with chemometrics was used to monitor the fermentation process and detect microvinifications and defective fermentations as an early detection system. In this study, sugar concentration was monitored with FTIR from the beginning to the end of the fermentation process to detect slower fermentation at the early stage. In another study, nitrate was monitored using ATR‐FTIR spectroscopy during plant growth (Ma et al. 2021). However, these studies comment on the present quality defect and are based on the analysis of the current sample. In this way, each process can be corrected.

In this study, FTIR spectra of the production steps, including conching, tempering, and cooling, were recorded to obtain a spectral pattern of chocolate production and to predict quality problems, such as physicochemical defects, and structural instability (fat and sugar bloom) in the end product. This new approach may be used as an early alert system in the industry to adjust process parameters, determine rework samples, and improve quality while reducing economic loss.

## 2 Materials and Methods

### 2.1 Materials

**FIGURE 1:**
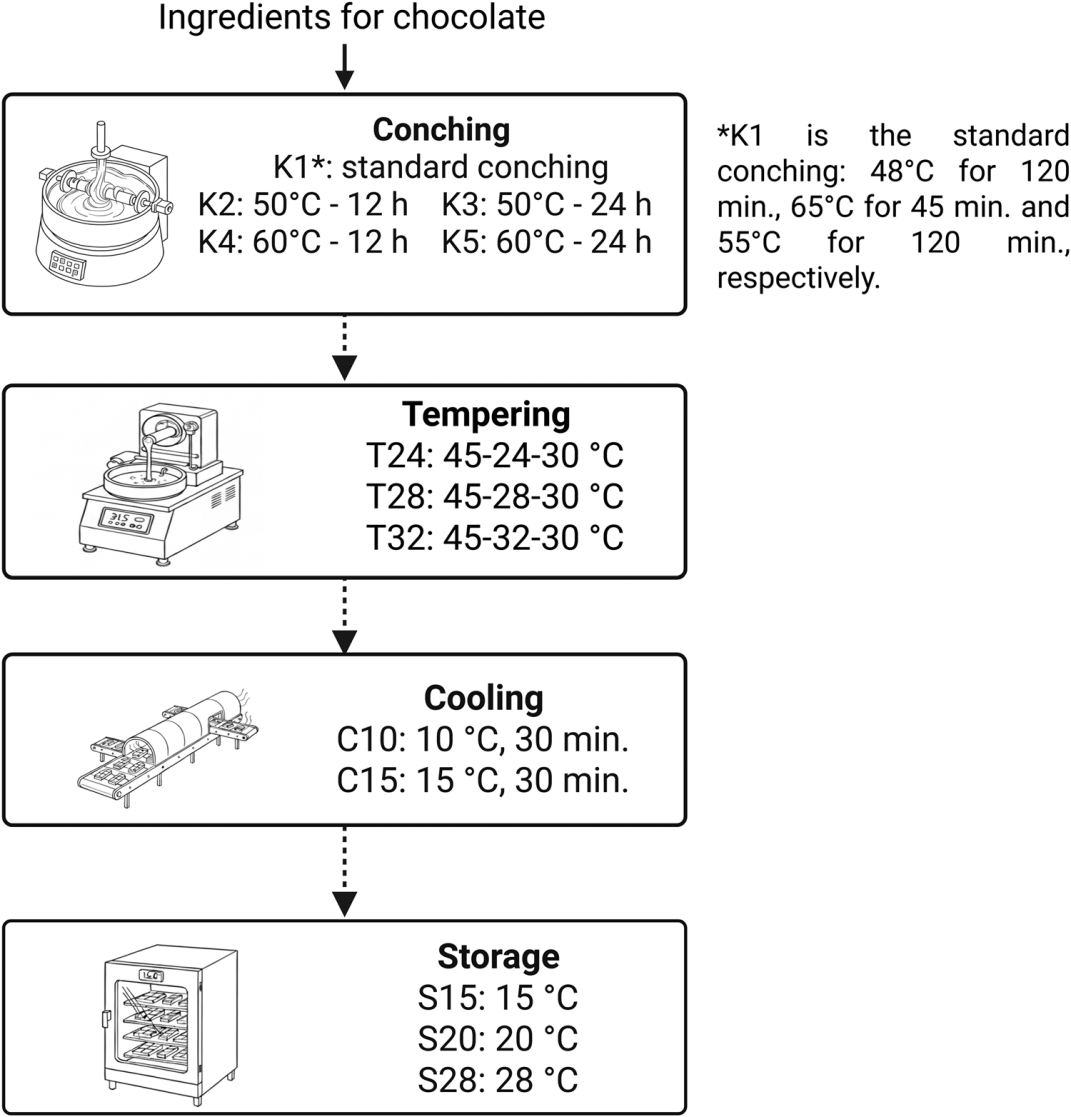
Chocolate production processes and parameters.

The chocolate was produced like in our previous study using the following ingredients: sucrose (Torku Gıda) (53%), cacao butter (Altınmarka, Beşel Gıda) (24%), fat milk powder (Altınmarka, Beşel Gıda), and natural cacao mass from Ghana origin (10% for each), dried milk powder (Altınmarka, Beşel Gıda) (2.41%), milk fat‐anhydrous (<1%), sunflower oil lecithin as an emulsifier (Brenntag Chemistry) (0.55%), aroma compound (Tito) (<1%), and table salt (Tito) (<1%), in accordance with the production process and parameters specified in Figure 1 (Küçükduman et al., 2026).

### 2.2 Chocolate Preparation and Storage

Natural cocoa mass is mixed in a heated chocolate mixer at 50°C for 40 min until a homogeneous paste is formed. The mixture, with an initial particle size greater than 500 µm, is transferred to rollers and refined until the final average particle size is reduced to the 20–25 µm range. Then, the moisture and odor of the chocolate paste are removed with hot air. Afterwards, sucrose (the average particle size of sugar is 0.464 mm) and other ingredients are added, and a conching process is applied at 30 rpm with different temperature and time parameters. All materials were conched at five different process parameters (K1–K5) as described in Figure 1 until mean particle size of samples less than 50 µm was reached. The particle size of each conched sample was measured using a laser diffraction particle size analyzer (Malvern Panalytical, Masterizer 3000, UK) like in the study of Koca (2011). The obtained samples were tempered in a tempering device (Pomati, T5, Italy) at three different temperatures (T24: 45–24–30°C, T28: 45–28–30°C, T32: 45–32–30°C), then poured into five different polycarbonate chocolate molds (6 cm × 12.60 cm), which were preheated in an oven at 30°C. They were then cooled in a refrigerated incubator (Nucleon, NSI 120) at two different temperatures (C10: 10°C and C15: 15°C) for 30 min. Sample temperatures were measured regularly using an infrared thermometer (Fluke, 59 MAX) throughout all intermediate processes. Final products were wrapped in aluminum foil and labeled. The quality parameters and defects of the products were examined during 8 months at three different storage temperatures (S15: 15°C, S20: 20°C, S28: 28°C).

### 2.3 Quality Analysis

To obtain defective samples, chocolates were stored at three different temperatures (15°C, 20°C, and 28°C). Texture, water activity, color analyses, and visual examination were performed on samples from the second, fourth, and eighth months.

#### 2.3.1 Texture Analysis

The method described by Mokbul et al. (2023) was adopted with slight modifications. Hardness analysis was performed using penetration testing with a texture analyzer (TA.XT Plus C, Stable Micro Systems Ltd., Nottingham, UK). A P/2 N needle‐tip texture probe was used in the analyses. The product height was 5 mm, and the penetration depth was 2 mm. The following parameters were taken into account during the test: the test speed was 0.5 mm/s, and the final speed was 10 mm/s. The test duration lasted approximately 1 min. The maximum penetration force (N) was recorded as the hardness of the chocolate. Analyses were performed in at least six replicates for each sample.

#### 2.3.2 Water Activity

The water activity of the chocolate samples was measured at 20°C with a water activity analyzer (ROTRONIC HP‐23) using 2 g of each grated sample (Konar 2013).

#### 2.3.3 Color Analysis

L* (brightness, 0–100), a* (redness–greenness), and b* (yellowness–blueness) values of chocolate samples were measured with a colorimeter (Konica Minolta, CM‐5, Japan). Surface bloom (fat and sugar bloom) in chocolate samples was obtained by measuring the L*, a*, and b* values with a colorimeter and calculating them according to the whiteness index (WI) equation specified in the following equation (Lohman and Hartel 1994):

(1) $$\mathrm{WI}=100-\sqrt{\left({\left(100-L^{*}\;\right)}^{2}+{\left(a^{*}\right)}^{2}+{\left(b^{*}\right)}^{2}\right)}$$

#### 2.3.4 Visual Examination of Chocolate Samples

Additional visual analyses were conducted to identify fat bloom and sugar bloom using the method described by Heuler et al. (2020) with slight modifications. To distinguish between fat and sugar bloom, samples with whiteness and surface changes were visually analyzed after incubation in an oven at 50°C for 30 min. If the whiteness on the top layer of the samples disappeared after the heat treatment, it was considered fat bloom; if it did not, it was considered sugar bloom.

### 2.4 Polarized Light Microscope Images

The samples were analyzed for changes in microscopic structure during storage using a polarized‐light microscope (Lonchampt and Hartel 2006). Before the imaging process, different parts of each chocolate sample (surface, edges, and bottom) were grinded with a knife. The grinded sample was placed between a microscope slide and cover slip. The images were taken of both unmelted and melted chocolates at 40°C to analyze fat and sugar bloom. The melting process was applied using the same method as in the visual examination above. Measurements were performed at 20× with a ZEISS AXIO Imager A2 polarized‐light microscope.

### 2.5 DSC Analysis

DSC analyses were conducted to identify the crystal structures of chocolate samples produced with five different conches and three different tempering parameters and cooled at 10°C 6 months after production. Measurements were performed using a Mettler Toledo DSC1 STAR System (Ohio, US) over the temperature range of 0–60°C, with a heating rate of 5°C/min and a nitrogen flow rate of 50 mL/min.

### 2.6 FTIR Analysis

FTIR spectra were obtained from all chocolate samples after each process (conching, tempering, and cooling) using an FTIR device with Nicolet iS10‐diamond ATR accessories (Thermo Scientific, USA) and a DTGS KBr detector at wavelengths between 4000 and 500 cm^−1^. Chocolate surfaces were placed on the ATR crystal, and measurements were taken. After the tempering process, liquid form of chocolate was dropped onto the ATR crystal. Each measurement was recorded with 64 spectra at a resolution of 4 by using the OMNIC Program (version 2.8).

Merging of FTIR spectra to obtain spectral pattern was performed with the changing regions in FTIR spectra within conching, tempering, and cooling processes. These regions were determined as described in our previous paper using partial least squares‐discriminant analysis (PLS‐DA) loading plots (Küçükduman et al. 2026). Approximately 11,000 lines of spectral patterns were obtained within the effective wavelength ranges, respectively: 1000–1800 and 2750–3750 cm^−1^ for conching; 500–1800 and 2750–3010 cm^−1^ for tempering; and 600–1800 and 3000–3750 cm^−1^ for cooling. At least three replicate spectra were obtained for each sample, and the averaged spectra were used for modeling. In the conching process, changes between 1000 and 1800 cm^−1^ wavenumbers indicate the ester group changes, whereas 2750–3750 cm^−1^ interval is related to water content and phenolic compound changes. On the other hand, tempering and cooling processes changed the crystal structure, which is obvious mainly in 500–1800 and 2750–3750 cm^−1^ wavenumbers. Detailed explanations were presented in previous study (Küçükduman et al. 2026).

### 2.7 Data Analysis

In this study, principal component analysis (PCA), an unsupervised data analysis technique, and PLS‐DA, a supervised data analysis technique, were used to analyze the quality parameters and FTIR spectra of chocolate samples. Data analyses were performed using Solo 9.3 software (Eigenvector Research Inc., Wenatchee, WA, USA).

The first PCA model was obtained from DSC analysis of chocolate samples produced and stored under different conditions (Section 2.1) to observe their effects on the crystal structure. In the model, 60 DSC diagrams were used. The number of PCs is 2, and preprocessing is autoscale.

To consider changes in sample within production parameters and storage conditions, three PCA models were constructed. Combined quality analyses data (color‐L*, a*, and b* value + WI + water activity + texture) of all samples, including newly produced (*t* = 0) and stored at 15°C, 20°C, and 28°C samples, were used for a 2, 4, and 8 months PCA model, respectively. In each model, 120 samples’ data were used, and autoscale preprocessing was applied. The number of PCs was 2.

For determination of physicochemical defects in newly produced chocolate (*t* = 0), samples were labeled with the aid of PCA method. For this purpose, uniform and physicochemical defective samples (improper water activity, color, WI, and hardness) were discriminated according to physicochemical analysis results. In the model, 30 samples (obtained from 5 different conching, 3 different tempering, and 2 different cooling parameters) were used. The number of PCs was 3, and autoscaling was used as preprocessing.

For future defect prediction (physicochemical defects and fat/sugar bloom as structural instability), PLS‐DA was employed. Three PLS‐DA models were obtained: one for physicochemical defective product prediction in new production (*t* = 0), one for fat bloom prediction, and one for sugar bloom prediction. To create the best model, the lowest RMSE values (RMSEC, RMSECV, and RMSEP) and the highest specificity and sensitivity values were selected for all PLS‐DA models.

For physicochemical defective products (improper water activity, color, WI, and hardness) prediction (*t* = 0), 59 spectral patterns were used for PLS‐DA, the mean‐center preprocessing method was applied, and LVs were set to 8. Calibration: Validation ratio was 3:1.

In visual tests, fat and sugar bloom were observed regularly in samples at all storage temperatures, with the first detection occurring at the end of the eighth month in samples at 15°C and 28°C. Each sample consisted of approximately 5 different molds (6 cm × 12.60 cm), each containing 15 pieces. Samples showing sugar or fat bloom in at least two pieces per mold were considered defective. Using spectral patterns derived from FTIR spectra obtained during the production of the chocolate samples, PLS‐DA modeling was performed on samples with fat and sugar bloom (defective) and on uniform samples during storage. In each model, 59 spectral patterns were used, and an autoscale preprocessing method was applied. LVs were adjusted to 14 and 13 for 15°C and 20°C, respectively. Calibration: Validation ratio was 2:1.

## 3 Results and Discussion

### 3.1 Spectral Pattern

**FIGURE 2:**
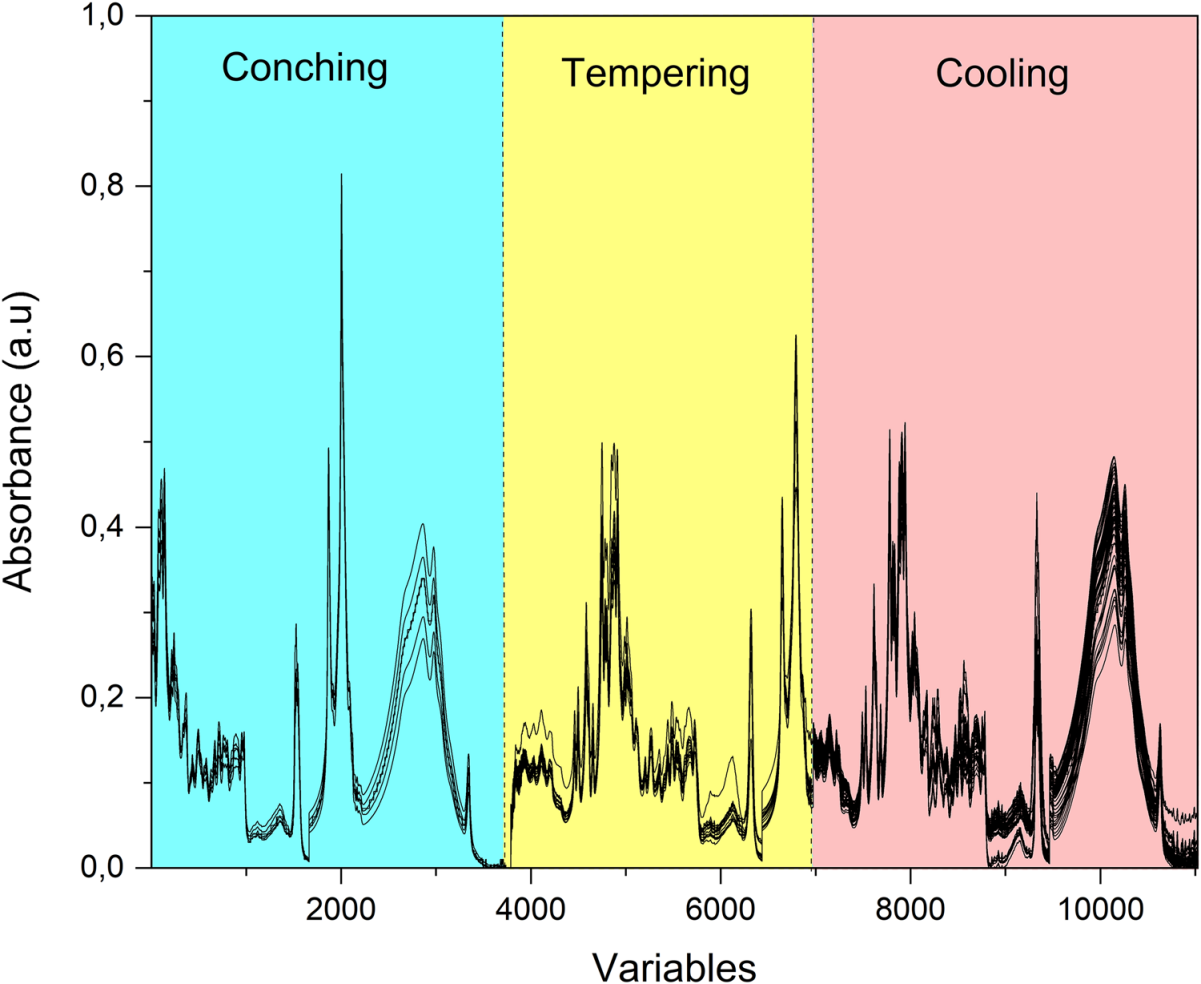
Spectral pattern of chocolate samples using FTIR spectra.

Each production step, including conching, tempering, and cooling, causes changes in FTIR spectra due to chemical variations during the processes. The conching process causes moisture reduction and aroma improvement in chocolate, whereas tempering and cooling processes are related to the crystal structure of cocoa butter. Wavenumber shifts in FTIR spectra were determined as described in our previous paper using PLS‐DA loading plots (Küçükduman et al. 2026). Then, these changing regions were combined to obtain a spectral pattern of chocolate production for use in future quality‐defect prediction models (Figure 2).

### 3.2 Quality Defects

Quality defects in chocolate can be classified into physicochemical defects and structural instability (fat bloom and sugar bloom). These defects were analyzed using several methods, as described below.

#### 3.2.1 Physicochemical Defects

**FIGURE 3:**
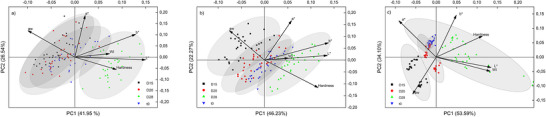
PCA graphs of differently processed chocolate samples stored during 2 months (a), 4 months (b), and 8 months (c).

In chocolate samples, most known quality defects, fat and sugar bloom, generally appear during the storage period. However, other textural quality defects appear quickly after production, depending on process parameters, and continue to deteriorate during shelf life. In this context, texture, color, WI, and water activity values were evaluated for new production and stored samples. Using the raw data from the analysis results (Tables S1–S4), the distribution of samples representing all production parameters was examined using PCA modeling. Figure 3 shows PCA graphs of new production samples and samples stored at 15°C, 20°C, and 28°C after 2 months (Figure 3a), after 4 months (Figure 3b), and after 8 months (Figure 3c). The model was constructed using texture, color, WI, and water activity values. It is clearly seen in the graphs that the variation between clusters with storage temperatures increases over time. Particularly, 28°C storage is entirely different from the other samples even after 2 months. On the other hand, samples stored at 20°C are quite similar to new production regardless of time. In the following section, each quality analysis was discussed.

##### 3.2.1.1 Texture

Chocolate texture plays an important role in consumer acceptance, as it reflects both the crystal structure and the mechanical integrity of the fat–particle network (Afoakwa et al. 2009b). Textural properties are affected by triglyceride polymorphism, microstructure, particle size distribution, and solid fat content (Nightingale et al. 2011).

The hardness of chocolate samples produced by different conching, tempering, cooling, and storage conditions (15°C, 20°C, and 28°C) is shown in Table S1. At the beginning of storage, hardness values ranged between approximately 5.3 and 7.5 N, depending on conching, tempering, and cooling conditions. Samples subjected to low temperature conching showed a decreasing trend in hardness during storage at 15°C, which can be result from high moisture uptake at low temperatures causing softer structure. In contrast, chocolates stored at 28°C showed a noticeable increase in hardness during storage of 8 months. Storage at 28°C increased hardness across all chocolate formulations. Initial values ranged from 5.3 to 7.5 N and increased to 8–12 N after 8 months, particularly in samples tempered at 28°C and 32°C. This hardening is linked to fat bloom formation, induced by the conversion from βV to βVI crystals and fat molecules reaching the surface at higher storage temperatures (Silva et al. 2017). Similar trends were observed in K2–K5 samples. This supports that storage temperature is the main factor affecting long‐term texture stability.

Other studies have reported similar results, showing that higher storage temperatures increase chocolate hardness (Nightingale et al. 2011). A study by Biczó et al. (2013) demonstrated that chocolate samples stored in a warm storage environment (22–28°C) for 8 months had hardness results that were approximately twice as high as those stored for one week. Furthermore, several studies reported polymorphic forms and crystallization kinetics, highlighting the critical role of Form V in achieving desirable texture and gloss (Afoakwa et al. 2008; Zhao et al. 2018). Moreover, storage‐induced polymorphic transitions, such as Form V–VI, were connected to texture degradation and fat bloom formation (Nightingale et al. 2012; Zhao et al. 2018). In addition, storage at low temperatures (6–12°C) preserved texture and minimized fat bloom, whereas high temperatures accelerated texture degradation (Andrae‐Nightingale et al. 2009; Hřivna et al. 2021).

Regardless of the storage temperature, for standard conched samples (K1), chocolates tempered at 24–28°C generally had a higher initial hardness value than those tempered at 32°C, especially when combined with lower cooling temperatures.

##### 3.2.1.2 Water Activity

Water activity is a critical property for the stability of chocolate quality, as it affects moisture migration, sugar bloom formation, and fat mobility within the chocolate matrix (Konar and Bingol 2019). Processes such as conching and tempering reduce particle interactions, affecting moisture‐related properties of chocolate (Afoakwa 2016; Glicerina et al. 2013; Konar and Bingol 2019; Toker et al. 2019). Conching time and temperature influence flavor and moisture‐related properties (Augusto and Bolini 2022; Villafuerte‐Carrillo et al. 2024). Moreover, moisture migration within chocolate matrices is influenced by processing parameters and storage conditions, with higher temperatures accelerating moisture transfer and quality degradation (Svanberg et al. 2012).

The water activity of chocolates produced under different conching and tempering conditions and stored at 15°C, 20°C, and 28°C over 8 months is presented in Table S2. During storage, an increase in water activity was observed across all formulations and processing conditions. At 15°C, water activity increased over time for all chocolate samples. The initial values ranged from 0.25 to 0.41, depending on the conching and tempering conditions. Chocolates processed under standard or low temperature conching (K1–K3) showed a significant increase in water activity, from 0.65 to 0.77, at the eighth month of storage. In contrast, samples processed under higher temperature conching (K4, K5) started with lower values and increased more slowly.

Samples tempered at 28°C and 32°C had lower water activity than those tempered at 24°C. This shows a more stable fat crystal network that limits moisture change in chocolate samples. At 20°C, the increase in water activity was more controlled than at 15°C, with values <0.55. At 28°C, water activity changed significantly, ranging from 0.38 to 0.47 at the eighth month of storage, lower than that at 15°C. This may be due to greater molecular mobility and moisture loss during storage at higher temperatures. Conching intensity stayed important at 28°C, with K4 and K5 samples having the lowest water activity. Tempering at 28°C and 32°C showed lower water activity than at 24°C, especially at the end of the storage period.

##### 3.2.1.3 Color and WI

The WI can be used as one of the quality parameters for chocolate, reflecting changes in color quality resulting from inadequate cooling or storage conditions. It indicates an overall shift from the original brown color, resulting in low WI, to a whitish color, resulting in high WI due to surface blooming (Briones and Aguilera 2005). It is calculated using L*, a*, and b* values in color analysis; however, because the average is measured by averaging parts of the chocolate surface, it leads to higher standard deviations that increase with blooming (Tables S3 and S4) (Quevedo et al. 2013). Moreover, the reason could be the nonhomogeneous formation of white spots on the surface of chocolate (Briones and Aguilera 2005). Lonchampt and Hartel (2006) also stated that the WI method for blooming characterization is not as reliable as visual examination.

Samples stored at 15°C and 20°C showed little change in WI and L*, whereas samples stored at 28°C showed higher results for both WI and L*, especially after 8 months of storage. Bui and Coad (2014) stated that storage time impacts mostly L* values (Table S3). Similarly, in a study by Machálková et al. (2015), storage at 30°C resulted in higher L* values. In contrast, storage at 6°C, 12°C, and 20°C did not significantly affect L* due to chocolate defects caused by blooming. For the a* and b* results, no considerable change was observed during the storage period.

Samples stored at 15°C showed lower L* values than those stored at higher temperatures. This can be attributed to the high moisture uptake of chocolate during low‐temperature storage. Similar findings were obtained by Lonchampt and Hartel (2006). In the study, a relationship was observed between higher moisture content and lower whiteness values in chocolates.

##### 3.2.1.4 Quality Prediction on Newly Produced Chocolate Samples

**FIGURE 4:**
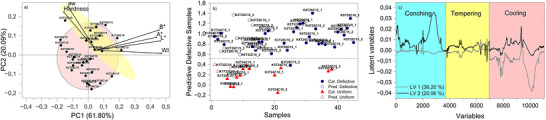
PCA graph of new chocolate production for labeling as uniform and defective (a), PLS‐DA score plot for physicochemically uniform and defective sample discrimination (b), and loading plot (c).

The spectral pattern derived from FTIR spectra was also used to predict physicochemical defects, including hardness, aw, L*, a*, b* values, and WI, in new production. For this modeling, the samples were first labeled uniform or defective for use in the PLS‐DA model. To discriminate the samples, a PCA model was constructed using combined quality parameters (hardness, aw, L*, a*, b* values, and WI) for newly produced chocolate samples. In the PCA biplot (Figure 4a), uniform samples are located at the intersection of the sample and variable clusters. In this cluster, standard production parameters and some other similar combinations are placed. The remaining samples were labeled defective. Figure 4b shows the PLS‐DA model for quality prediction in newly produced chocolate samples from FTIR spectra recorded during production. Results showed that physicochemically defective and uniform samples could be predicted with 100% sensitivity and specificity even at the early production step (Table 1). Score values of defective samples in Figure 4a exhibit significant deviations from the uniform samples’ score values, which include water activity, color, WI, and hardness values. On the other hand, K1, K4 conched and 28–32°C tempered samples are found in uniform samples. The reason may be the similarities in moisture and particle size between the K1 and K4 conched samples (Table S5). Furthermore, 28°C is known as the optimal tempering temperature (Windhab 2017). Moreover, in our previous study, 28°C and 32°C tempering similarity was shown in models (Küçükduman et al. 2026). Glicerina et al. (2015) showed that the final quality of chocolate depends strongly on its microstructure and rheology. In the study, the effects of each process (refining, conching, and tempering) on the rheology and microstructure were shown, similar to our study. In Figure 4b, the PLS‐DA model showed high sensitivity and specificity in discriminating between physicochemically uniform and defective samples (Table 1).

**TABLE 1:** Partial least squares‐discriminant analysis (PLS‐DA) models’ performance parameters.

Newly produced chocolate								
Calibration results			Cross validation results			Prediction results		
**Class**:	**TPR**	**FPR**	**Class**:	**TPR**	**FPR**	**Class**:	**TPR**	**FPR**
**Uniform**	0.97	0	**Uniform**	0.91	0.23	**Uniform**	1	0
**Defective**	1	0.03	**Defective**	0.77	0.09	**Defective**	1	0

**Defective and uniform sample separation at 15°C (sugar bloom)**								
**Calibration results**			**Cross validation results**			**Prediction results**		
**Class**:	**TPR**	**FPR**	**Class**:	**TPR**	**FPR**	**Class**:	**TPR**	**FPR**
**Uniform**	1	0	**Uniform**	0.80	0.17	**Uniform**	1	0
**Defective**	1	0	**Defective**	0.83	0.20	**Defective**	1	0

**Defective and uniform sample separation at 20°C (fat bloom)**								
**Calibration results**			**Cross validation results**			**Prediction results**		
**Class**:	**TPR**	**FPR**	**Class**:	**TPR**	**FPR**	**Class**:	**TPR**	**FPR**
**Uniform**	1	0	**Uniform**	0.82	0.27	**Uniform**	1	0
**Defective**	1	0	**Defective**	0.73	0.18	**Defective**	1	0

Abbreviations: FPR, false positive rate; TPR, true positive rate.

#### 3.2.2 Fat and Sugar Bloom Defects

In high‐temperature storage, almost all samples showed fat bloom regardless of the process parameters. Delbaere et al. (2016) also showed that fat bloom occurs at poorly controlled storage temperatures, even when chocolates are produced under optimal tempering and cooling parameters. Therefore, a 20°C storage temperature was used to detect fat bloom depending on the processing parameters. Fat bloom appeared on the chocolate surface after 4 months at 20°C storage, whereas sugar bloom appeared after 8 months at 15°C storage. To evaluate fat bloom and sugar bloom defects, several analyses were conducted. Polarized light microscope, DSC, WI, and visual test results were evaluated for that.

##### 3.2.2.1 Polarized Light Microscope

**FIGURE 5:**
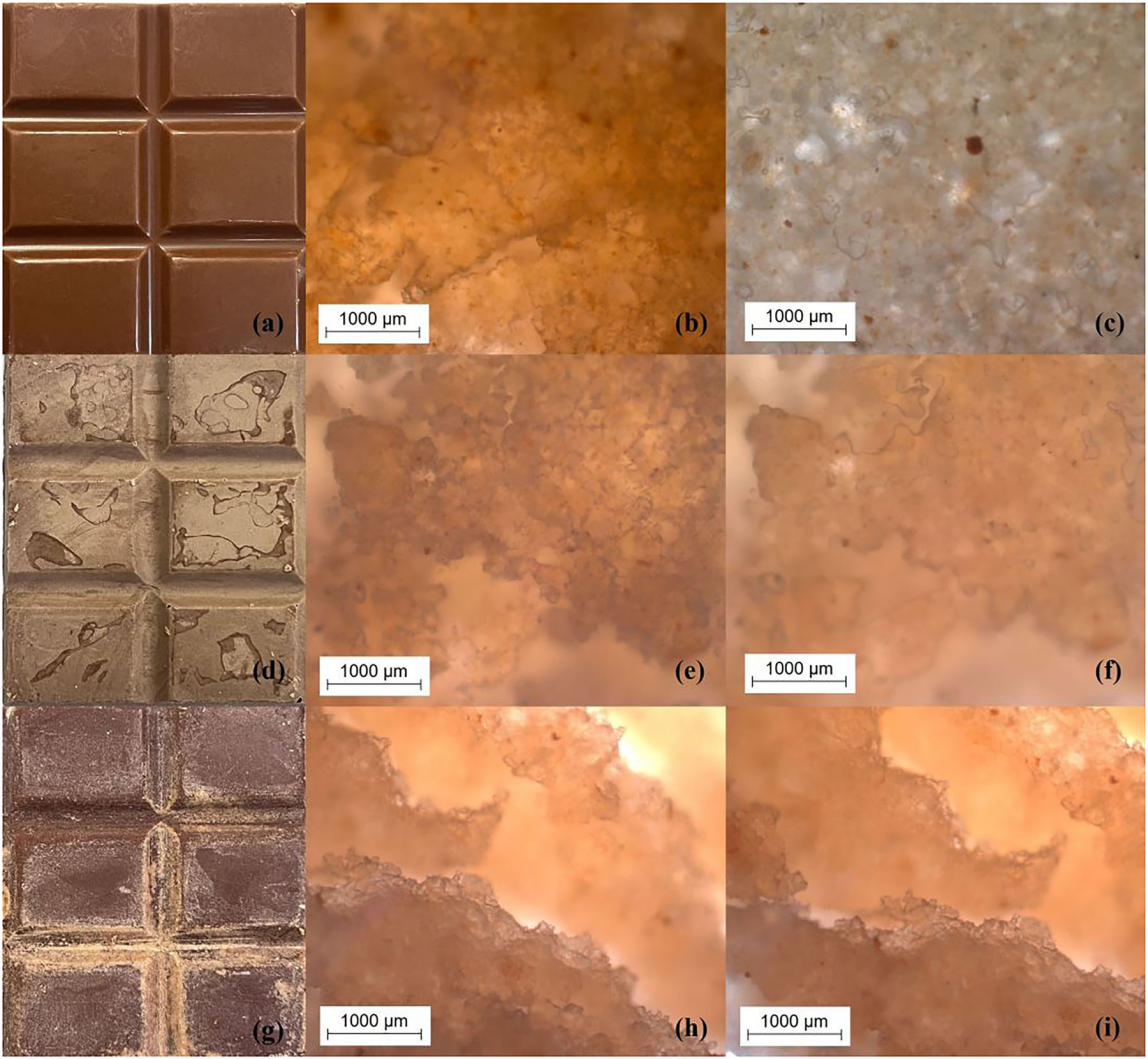
The appearance of standard sample (a), polarized light microscopy images for unmelted (b), and melted sample (c); the appearance of fat bloom at 28°C (d), polarized light microscopy images for unmelted (e), and melted sample (f); and the appearance of sugar bloom at 15°C (g), polarized light microscopy images for unmelted (h), and melted sample (i).

Because polarized light microscopy allows observation of crystal structure, defective samples were analyzed under a light microscope. In the microscope images, crystal structures indicate the fat or sugar bloom samples. A melting process was done at 50°C to separate them. If crystal structures disappear, it is labeled a fat bloom. If it remains, it is labeled a sugar bloom. The standard (K1T24S10) and defective samples, namely, sugar‐ and fat‐bloom chocolates (8‐month samples at 15°C and 28°C, respectively), were computed both in the unmelted and melted states. The appearance and light microscope images of standard chocolate are presented in Figure 5a–c, and the ones that have fat bloom and sugar bloom are presented in Figure 5d–i, respectively. The well‐conched and tempered chocolate showed that the fat crystals are homogeneously dispersed, as seen in Figure 5b,c. A crystalline structure was observed in the fat bloom sample, which is related to high‐temperature storage (28°C). This correlates with the presence of efflorescence in the samples and the high lightness (L*) and hardness values.

Furthermore, the microstructural observations clearly confirmed the DSC results. The chocolates stored at 28°C showed a broader melting enthalpy with a secondary peak, displaying the formation of the more stable form (βVI) of cocoa butter. This is commonly correlated with fat bloom formation. The polarized light microscopy image also confirms the crystal structure of fat, which disappeared after melting at 50°C (Figure 5e,f).

Sugar bloom is clearly visible in both the visual appearance and microscope images in Figure 5g–i. The microstructural images show sharp, rough sugar crystals in chocolate that remained unchanged after melting, indicating sugar bloom.

##### 3.2.2.2 Differential Scanning Calorimetry

The formation of fat bloom in chocolate products is strongly linked to melting characteristics obtained from polymorphic transitions in cocoa butter crystals. Literature reports indicate that the βIV, βV, and βVI crystal forms typically melt at around 28.7°C, 32.4°C, and 35.5°C, respectively (Afoakwa et al. 2009a; Konar et al. 2017).

**FIGURE 6:**
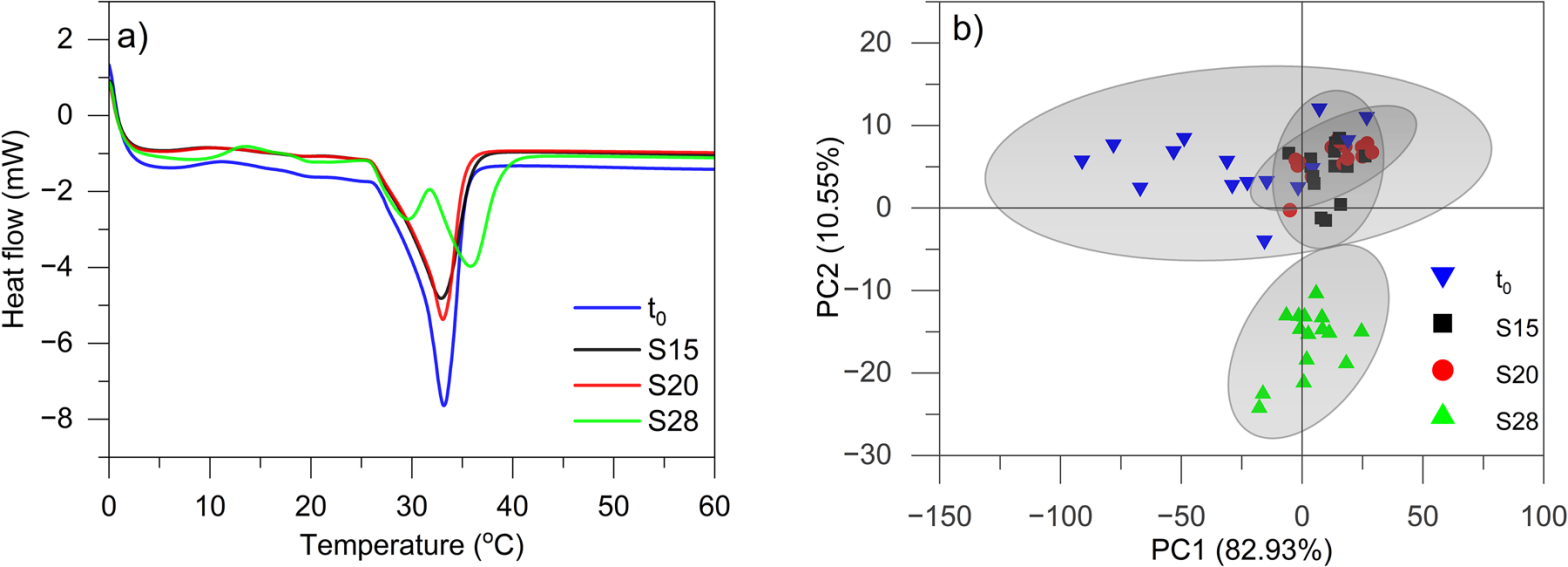
Average DSC thermograms of differently conched, tempered, and cooled chocolate samples stored at various temperatures (a); DSC thermogram PCA score plot (b).

Both storage temperature and moisture content play critical roles in these transformations, with higher temperatures promoting faster polymorphic reorganization (Nightingale et al. 2011; Škrabal et al. 2019). As shown in Figure 6a, all chocolate samples exhibited a major endothermic melting peak between 30°C and 40°C, characteristic of cocoa butter crystals in chocolate (Torbica et al. 2014). After production, the samples demonstrated a sharp melting endotherm, indicating a uniform crystal arrangement. During storage, especially at 28°C, the melting curve became broader, and an additional peak appeared at 36°C. This property has been associated with the formation of the βVI crystal form and regarded as a thermal indicator of fat bloom development (Silva et al. 2017). A broader, more complex melting behavior was observed after storage at 28°C. This structural change indicates a polymorphic transformation and crystal growth during storage at higher temperatures, as reported previously (Jin and Hartel 2020; Škrabal et al. 2019). This thermal behavior has been linked to a higher risk of fat bloom at warmer storage temperatures (Depypere et al. 2009; Machálková et al. 2015).

Overall, the DSC data indicate that higher storage temperatures accelerate the reorganization of the fat crystal network, promoting polymorphic instability and increasing the susceptibility to quality defects in chocolate products, such as fat bloom. Similar to these results, PCA (Figure 6b) clearly separated samples stored at 28°C from those stored at lower temperatures, indicating structural changes at high temperatures.

##### 3.2.2.3 Whiteness Index

The WI is an important quality parameter not only for quality assessment but also for fat bloom identification. The fat bloom‐WI relation was explained above in Section 3.2.1.3.

##### 3.2.2.4 Fat and Sugar Bloom Prediction Models on Stored Chocolate Samples

The generated spectral pattern of chocolate production (combined FTIR spectra) was used to determine future fat and sugar bloom defects in this study. Produced samples were stored under different conditions (15°C, 20°C, and 28°C) to obtain defective samples. However, high‐temperature storage (28°C) causes fat bloom in almost all samples within 2 months, regardless of production parameters, including conching, tempering, and cooling (Figure 3a). Ali et al. (2001) also showed that 30°C storage of chocolates accelerated fat migration and bloom formation in all samples. This result is obvious in WI values (Table S4) and visual appearance tests in this study. As a result, 20°C and 15°C storage samples were used to discriminate between defective and uniform samples. Results showed that fat bloom appeared in samples stored at 20°C, whereas sugar bloom appeared in samples stored at 15°C. Therefore, two prediction models were developed for fat and sugar bloom separately, which appear clearly after 8 months.

**FIGURE 7:**
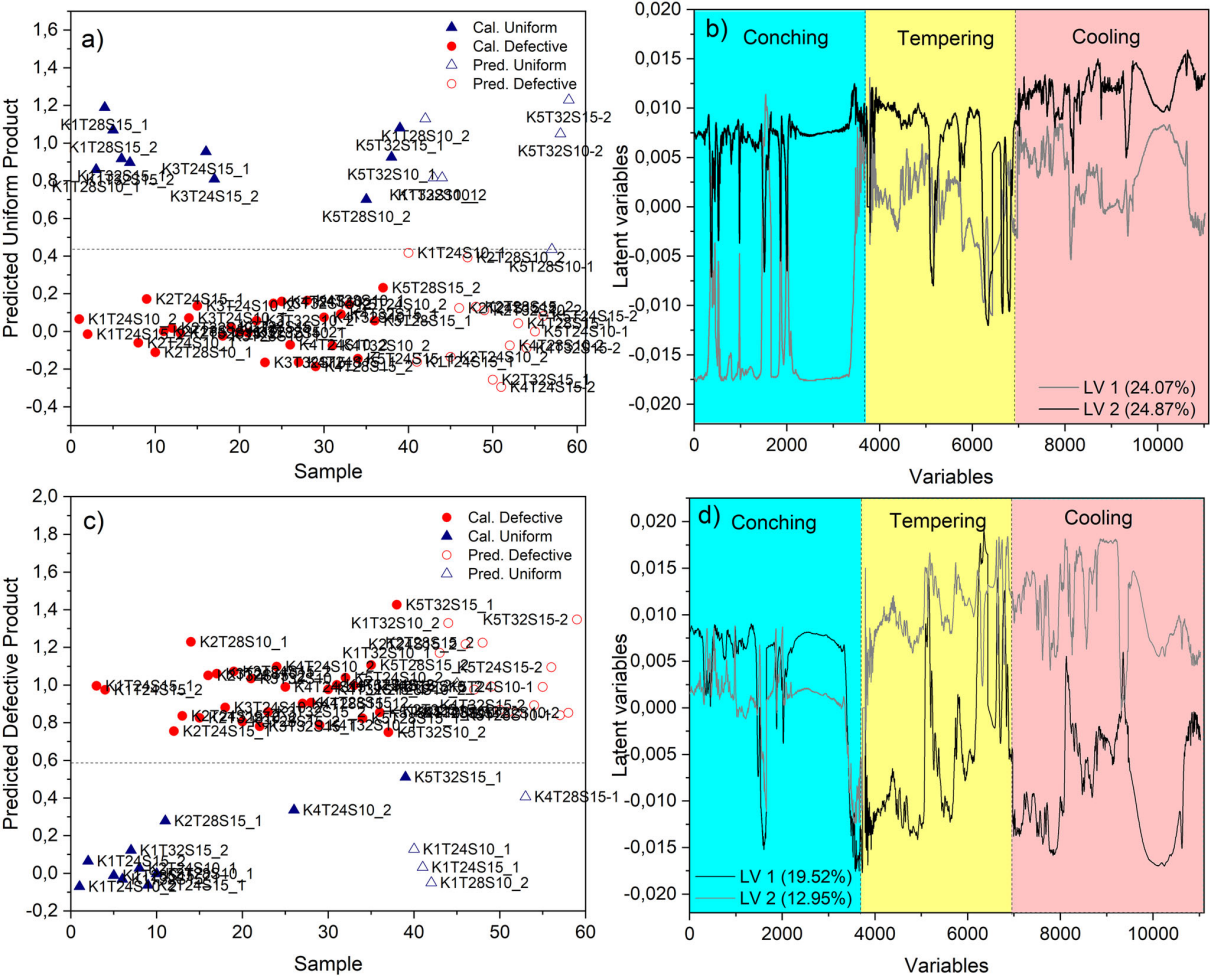
PLS‐DA score plot constructed by spectral pattern for fat bloom prediction (a) and loading plot (b); and for sugar bloom prediction (c) and loading plot (d).

To evaluate the fat bloom caused by improper production parameters, 20°C stored samples were used for chemometric modeling. To classify the data set as defective (fat bloom) or uniform, visual appearance tests were performed as described above. According to this classification, we obtained 44 samples as defective and 15 as uniform. Figure 7a,b shows the PLS‐DA model for fat bloom and uniform sample prediction model and loading graph. Regarding the results, FTIR spectra recorded during production could be used to address future fat bloom problems that may occur after 8 months. In the model, it was observed that the industrial conching process (K1) produced more uniform samples, whereas the other conching processes produced defective samples due to high temperatures or long times. This may be directly attributed to the particle size of the chocolate. A study showed that smaller particles reduce fat bloom due to hydrophobic forces within capillary channels, whereas larger particles accelerate fat migration through the surface. On the other hand, hardness showed a reverse relationship with particle size during bloom formation (Afoakwa et al. 2009a). Furthermore, unsuitable process parameters, such as under‐tempering (T45‐24‐32), caused fat bloom in all chocolate samples stored at 20°C, regardless of the conching process. Similar findings were obtained by Lonchampt and Hartel (2006). Furthermore, among the samples conched with industrial parameters (K1), 28°C tempering and 10°C and 15°C cooling parameters yield uniform samples. Cooling at 10°C and 15°C gave results similar to those in the study by Keijbets et al. (2010). In the study, it was shown that surface adhesion and hardness values dramatically decreased after 15°C cooling. Furthermore, higher cooling temperatures produced larger crystals, whereas lower cooling temperatures (0–10°C) produced smaller crystals in the study.

Sugar bloom defects just appeared in samples stored at 15°C. The main reason is high‐humidity storage. When the surface temperature of chocolate drops to its dew point, water condenses on the surface (Talbot 2014). Therefore, this sample group was used for chemometric modeling to predict the sugar bloom defect. To classify the data set as defective (sugar bloom) or uniform, visual appearance tests were performed. According to this classification, we obtained 43 samples as defective and 16 samples as uniform. Figure 7c,d shows the PLS‐DA model for sugar bloom and the uniform sample prediction model and loading graph. The model parameters are shown in Table 1 for the determination of defective samples. Uniform and defective samples can be separated with 100% specificity and sensitivity, as indicated by the calibration and prediction results of both models.

In the model, most of the uniform samples belong to K1 and K5 conching parameters. This can be explained by the lower water activity values of K1 and K5 chocolate samples (Table S2).

## 4 Conclusion

In this study, the detection of major structural instability problems (fat and sugar blooming) in chocolate before they become visible was successfully achieved using the PLS‐DA model. Samples were produced with different parameters to generate defective samples, and their FTIR spectra were taken after each process to create a sample‐specific spectral pattern (sample fingerprint). Defective samples were separated from uniform samples by establishing a PLS‐DA model using chemical analysis results obtained at *t* = 0. In addition, evaluation of defective samples formed during storage was also performed using classical chemical analyses. Differences between samples were observed using different chemometric models (PCA, PLS‐DA). In the 15°C and 20°C samples, sugar and fat blooming were detected under different conditions, respectively, enabling the separation of defective and uniform samples. In the food industry, detecting defective end products during production can reintroduce them into production, ensuring consumer access to quality products and reducing economic losses for producers. This research can be used as a pioneering tool to prevent potential defective products during the production of different food matrices in the food industry, thereby ensuring economic, consumer‐oriented, and sustainable production.

## Author Contributions

**Yağmur Küçükduman**: conceptualization, data curation, validation, methodology, visualization, formal analysis, investigation, writing – original draft preparation. **İkra Doğa Korkmaz**: formal analysis and investigation. **Hüseyin Güray Çiftçi**: formal analysis. **Gökhan İpkin**: formal analysis. **Sinem Argün**: formal analysis, writing – review and editing. **Zeynep Pınar Kara**: formal analysis. **Gonca Bilge Özel**: conceptualization, data curation, validation, methodology, investigation, writing – original draft preparation, supervision, project administration, funding acquisition. **Özge Taştan Ülkü**: writing – original draft preparation. **Ömer Said Toker**: supervision. **Zeynep Mutlu**: formal analysis.

## Funding

This study was supported by Scientific and Technological Research Council of Turkey (TUBITAK) under the Grant Number 223O050.

## Conflicts of Interest

The authors declare no conflicts of interest.

## Supporting information



Table S1. Hardness changes of chocolate samples within time. Table S2. Water activity changes of chocolate samples. Table S3. Color changes of chocolate samples within time. Table S4. WI changes of chocolate samples within time. Table S5. Moisture, water activity, and particle size values of variously conched samples after conching process.

## Data Availability

Data will be made available on request.
