# Supplementary material for: Spectral Pattern of Chocolate Production: Early Detection of Quality Problems
Source: J Food Sci. 2026 Jul 20;91(7):e71269. doi: 10.1111/1750-3841.71269 (PMC13383598; doi:10.1111/1750-3841.71269)
Supplement: Supplementary file 1 — Table S1. Hardness changes of chocolate samples within time. Table S2. Water activity changes of chocolate samples. Table S3. Color changes of chocolate samples within time. Table S4. WI changes of chocolate samples within time. Table S5. Moisture, water activity, and particle size values of variously conched samples after conching process. [file JFDS-91-0-s001.zip › jfds71269-sup-0001-TableS1.docx]

| Sample | Hardness (N) | | | |  | Sample | Hardness (N) | | | |
| --- | --- | --- | --- | --- | --- | --- | --- | --- | --- | --- |
|  | t=0 | t=2 | t=4 | t=8 |  |  | t=0 | t=2 | t=4 | t=8 |
| **K1T24S10D15** | 6.33±0.31 | 5.17±0.13 | 4.55±0.09 | 3.63±0.07 |  | **K3T28S15D20** | 7.13±0.27 | 5.23±0.16 | 6.27±0.13 | 5.84±0.37 |
| **K1T24S15D15** | 5.77±0.23 | 5.3±0.10 | 4.47±0.36 | 4.56±0.09 |  | **K3T32S10D20** | 7.12±0.22 | 4.07±0.20 | 5.38±0.06 | 6.53±0.26 |
| **K1T28S10D15** | 6.21±0.14 | 5.34±0.13 | 4.47±0.17 | 3.45±0.17 |  | **K3T32S15D20** | 7.45±0.15 | 5.77±0.19 | 6.29±0.20 | 6.06±0.17 |
| **K1T28S15D15** | 5.82±0.17 | 5.30±0.17 | 4.19±0.14 | 3.31±0.12 |  | **K4T24S10D20** | 5.74±0.17 | 5.04±0.15 | 4.60±0.11 | 5.84±0.09 |
| **K1T32S10D15** | 5.90±0.21 | 4.73±0.14 | 3.65±0.09 | 3.08±0.18 |  | **K4T24S15D20** | 6.67±0.40 | 5.64±0.15 | 6.08±0.29 | 6.70±0.16 |
| **K1T32S15D15** | 6.28±0.21 | 5.41±0.13 | 4.40±0.14 | 3.30±0.06 |  | **K4T28S10D20** | 5.67±0.20 | 5.33±0.15 | 5.52±0.11 | 6.15±0.04 |
| **K2T24S10D15** | 5.54±0.20 | 4.67±0.17 | 3.19±0.3 | 3.34±0.02 |  | **K4T28S15D20** | 5.68±0.08 | 5.45±0.12 | 5.12±0.13 | 6.07±0.19 |
| **K2T24S15D15** | 5.66±0.08 | 4.96±0.13 | 3.55±0.25 | 3.20±0.10 |  | **K4T32S10D20** | 5.67±0.17 | 4.89±0.15 | 5.64±0.09 | 6.20±0.17 |
| **K2T28S10D15** | 5.82±0.16 | 4.58±0.38 | 3.75±0.04 | 3.14±0.05 |  | **K4T32S15D20** | 6.66±0.18 | 5.69±0.26 | 6.05±0.10 | 6.31±0.10 |
| **K2T28S15D15** | 5.96±0.11 | 4.53±0.18 | 3.66±0.11 | 2.50±0.04 |  | **K5T24S10D20** | 6.15±0.59 | 5.26±0.09 | 5.72±0.22 | 5.57±0.10 |
| **K2T32S10D15** | 5.81±0.07 | 3.85±0.08 | 3.40±0.15 | 2.15±0.04 |  | **K5T24S15D20** | 5.33±0.31 | 5.14±0.15 | 5.29±0.14 | 6.09±0.05 |
| **K2T32S15D15** | 6.05±0.09 | 3.88±0.16 | 3.50±0.29 | 2.06±0.02 |  | **K5T28S10D20** | 5.40±0.15 | 5.07±0.03 | 5.56±0.14 | 5.95±0.14 |
| **K3T24S10D15** | 6.21±0.41 | 2.84±0.12 | 2.63±0.14 | 4.10±0.11 |  | **K5T28S15D20** | 5.82±0.25 | 5.34±0.10 | 5.51±0.11 | 6.08±0.09 |
| **K3T24S15D15** | 6.22±0.09 | 3.37±0.22 | 2.70±0.11 | 3.33±0.06 |  | **K5T32S10D20** | 5.86±0.13 | 5.39±0.16 | 5.97±0.10 | 6.20±0.15 |
| **K3T28S10D15** | 6.47±0.25 | 3.78±0.20 | 2.60±0.13 | 3.16±0.10 |  | **K5T32S15D20** | 5.94±0.16 | 5.23±0.43 | 5.74±0.32 | 6.44±0.12 |
| **K3T28S15D15** | 7.13±0.27 | 3.49±0.24 | 3.35±0.32 | 3.45±0.33 |  | **K1T24S10D28** | 6.33±0.31 | 5.35±0.08 | 7.97±0.35 | 9.48±0.68 |
| **K3T32S10D15** | 7.12±0.22 | 3.82±0.18 | 2.83±0.12 | 2.78±0.05 |  | **K1T24S15D28** | 5.77±0.23 | 4.86±0.15 | 8.08±0.20 | 9.78±0.36 |
| **K3T32S15D15** | 7.45±0.15 | 4.25±0.10 | 3.55±0.16 | 3.73±0.29 |  | **K1T28S10D28** | 6.21±0.14 | 6.78±0.33 | 9.13±0.16 | 10.68±0.59 |
| **K4T24S10D15** | 5.74±0.17 | 4.16±0.07 | 2.83±0.10 | 3.20±0.09 |  | **K1T28S15D28** | 5.82±0.17 | 7.01±0.14 | 9.80±0.87 | 11.41±0.73 |
| **K4T24S15D15** | 6.67±0.40 | 3.93±0.17 | 3.02±0.13 | 3.53±0.19 |  | **K1T32S10D28** | 5.90±0.21 | 7.62±0.16 | 10.69±0.17 | 11.55±0.42 |
| **K4T28S10D15** | 5.67±0.20 | 4.53±0.10 | 3.08±0.29 | 3.20±0.14 |  | **K1T32S15D28** | 6.28±0.21 | 6.58±0.18 | 10.67±0.31 | 12.52±0.35 |
| **K4T28S15D15** | 5.68±0.08 | 4.25±0.13 | 3.15±0.19 | 3.44±0.24 |  | **K2T24S10D28** | 5.54±0.20 | 5.15±0.29 | 6.49±0.21 | 9.57±0.11 |
| **K4T32S10D15** | 5.67±0.17 | 3.34±0.08 | 2.34±0.08 | 2.82±0.05 |  | **K2T24S15D28** | 5.66±0.08 | 5.93±0.10 | 6.28±0.08 | 9.69±0.29 |
| **K4T32S15D15** | 6.66±0.18 | 4.46±0.09 | 2.50±0.24 | 2.89±0.09 |  | **K2T28S10D28** | 5.82±0.16 | 6.60±0.37 | 8.65±0.52 | 8.32±0.74 |
| **K5T24S10D15** | 6.15±0.59 | 4.38±0.08 | 3.21±0.13 | 3.71±0.13 |  | **K2T28S15D28** | 5.96±0.11 | 7.45±0.37 | 8.10±0.39 | 10.69±0.67 |
| **K5T24S15D15** | 5.33±0.31 | 3.70±0.14 | 2.95±0.17 | 2.59±0.11 |  | **K2T32S10D28** | 5.81±0.07 | 6.35±0.34 | 8.47±0.27 | 9.92±0.60 |
| **K5T28S10D15** | 5.40±0.15 | 3.95±0.10 | 3.93±0.09 | 3.10±0.21 |  | **K2T32S15D28** | 6.05±0.09 | 7.30±0.20 | 7.87±0.09 | 8.98±0.67 |
| **K5T28S15D15** | 5.82±0.25 | 4.00±0.10 | 3.50±0.09 | 2.90±0.20 |  | **K3T24S10D28** | 6.21±0.41 | 6.58±0.18 | 5.97±0.11 | 5.87±0.29 |
| **K5T32S10D15** | 5.86±0.13 | 4.03±0.14 | 3.47±0.18 | 3.02±0.10 |  | **K3T24S15D28** | 6.22±0.09 | 6.44±0.14 | 7.48±0.16 | 7.62±0.58 |
| **K5T32S15D15** | 5.94±0.16 | 4.13±0.18 | 3.24±0.15 | 3.57±0.16 |  | **K3T28S10D28** | 6.47±0.25 | 7.47±0.23 | 8.77±0.80 | 6.90±0.41 |
| **K1T24S10D20** | 6.33±0.31 | 3.62±0.14 | 5.86±0.06 | 6.40±0.12 |  | **K3T28S15D28** | 7.13±0.27 | 7.36±0.10 | 9.34±0.13 | 9.43±0.71 |
| **K1T24S15D20** | 5.77±0.23 | 4.40±0.14 | 5.48±0.12 | 6.11±0.13 |  | **K3T32S10D28** | 7.12±0.22 | 7.94±0.17 | 9.88±0.25 | 10.11±0.61 |
| **K1T28S10D20** | 6.21±0.14 | 4.14±0.13 | 5.47±0.15 | 5.60±0.07 |  | **K3T32S15D28** | 7.45±0.15 | 9.23±0.13 | 9.07±0.55 | 11.04±0.34 |
| **K1T28S15D20** | 5.82±0.17 | 4.66±0.29 | 6.06±0.08 | 6.71±0.13 |  | **K4T24S10D28** | 5.74±0.17 | 5.64±0.15 | 7.63±0.19 | 7.91±0.40 |
| **K1T32S10D20** | 5.90±0.21 | 4.59±0.21 | 5.72±0.16 | 6.30±0.16 |  | **K4T24S15D28** | 6.67±0.40 | 5.63±0.53 | 7.32±0.08 | 8.94±0.14 |
| **K1T32S15D20** | 6.28±0.21 | 4.58±0.21 | 5.33±0.13 | 6.78±0.16 |  | **K4T28S10D28** | 5.67±0.20 | 6.15±0.32 | 8.13±0.17 | 9.69±0.32 |
| **K2T24S10D20** | 5.54±0.20 | 4.11±0.28 | 5.15±0.10 | 6.02±0.17 |  | **K4T28S15D28** | 5.68±0.08 | 5.44±0.07 | 7.40±0.28 | 8.30±0.39 |
| **K2T24S15D20** | 5.66±0.08 | 4.28±0.09 | 5.48±0.21 | 5.58±0.09 |  | **K4T32S10D28** | 5.67±0.17 | 6.45±0.10 | 7.65±0.40 | 9.86±0.12 |
| **K2T28S10D20** | 5.82±0.16 | 3.73±0.21 | 5.49±0.13 | 5.75±0.18 |  | **K4T32S15D28** | 6.66±0.18 | 7.19±0.16 | 7.96±0.72 | 10.55±0.21 |
| **K2T28S15D20** | 5.96±0.11 | 4.61±0.15 | 5.67±0.15 | 5.69±0.14 |  | **K5T24S10D28** | 6.15±0.59 | 4.52±0.31 | 6.78±0.32 | 7.15±0.30 |
| **K2T32S10D20** | 5.81±0.07 | 4.47±0.13 | 5.68±0.14 | 5.87±0.06 |  | **K5T24S15D28** | 5.33±0.31 | 5.02±0.20 | 7.59±0.24 | 8.35±0.35 |
| **K2T32S15D20** | 6.05±0.09 | 4.23±0.23 | 5.72±0.19 | 6.28±0.14 |  | **K5T28S10D28** | 5.40±0.15 | 6.43±0.22 | 5.42±0.16 | 8.93±0.37 |
| **K3T24S10D20** | 6.21±0.41 | 5.03±0.22 | 5.35±0.13 | 5.63±0.16 |  | **K5T28S15D28** | 5.82±0.25 | 5.66±0.10 | 7.47±0.29 | 9.19±0.31 |
| **K3T24S15D20** | 6.22±0.09 | 4.91±0.08 | 5.67±0.26 | 5.71±0.09 |  | **K5T32S10D28** | 5.86±0.13 | 6.06±0.09 | 8.07±0.37 | 8.64±0.41 |
| **K3T28S10D20** | 6.47±0.25 | 4.34±0.13 | 5.30±0.20 | 6.10±0.12 |  | **K5T32S15D28** | 5.94±0.16 | 7.75±0.09 | 8.78±0.77 | 10.6±0.37 |
