# Supplementary material for: Spectral Pattern of Chocolate Production: Early Detection of Quality Problems
Source: J Food Sci. 2026 Jul 20;91(7):e71269. doi: 10.1111/1750-3841.71269 (PMC13383598; doi:10.1111/1750-3841.71269)
Supplement: Supplementary file 1 — Table S1. Hardness changes of chocolate samples within time. Table S2. Water activity changes of chocolate samples. Table S3. Color changes of chocolate samples within time. Table S4. WI changes of chocolate samples within time. Table S5. Moisture, water activity, and particle size values of variously conched samples after conching process. [file JFDS-91-0-s001.zip › jfds71269-sup-0001-TableS2.docx]

| Sample | Water Activity | | | |  | Sample | Water Activity | | | |
| --- | --- | --- | --- | --- | --- | --- | --- | --- | --- | --- |
|  | t=0 | t=2 | t=4 | t=8 |  |  | t=0 | t=2 | t=4 | t=8 |
| **K1T24S10D15** | 0.30±0.00 | 0.44±0.02 | 0.48±0.00 | 0.65±0.00 |  | **K3T28S15D20** | 0.36±0.01 | 0.30±0.01 | 0.42±0.00 | 0.53±0.00 |
| **K1T24S15D15** | 0.30±0.01 | 0.42±0.00 | 0.48±0.01 | 0.66±0.00 |  | **K3T32S10D20** | 0.35±0.00 | 0.31±0.00 | 0.39±0.01 | 0.55±0.01 |
| **K1T28S10D15** | 0.38±0.02 | 0.37±0.00 | 0.47±0.01 | 0.65±0.00 |  | **K3T32S15D20** | 0.38±0.01 | 0.32±0.00 | 0.45±0.01 | 0.55±0.00 |
| **K1T28S15D15** | 0.41±0.01 | 0.40±0.00 | 0.49±0.00 | 0.66±0.00 |  | **K4T24S10D20** | 0.34±0.01 | 0.34±0.00 | 0.50±0.00 | 0.52±0.02 |
| **K1T32S10D15** | 0.34±0.00 | 0.41±0.01 | 0.47±0.03 | 0.67±0.00 |  | **K4T24S15D20** | 0.34±0.00 | 0.34±0.00 | 0.53±0.01 | 0.51±0.00 |
| **K1T32S15D15** | 0.35±0.02 | 0.41±0.00 | 0.48±0.00 | 0.69±0.00 |  | **K4T28S10D20** | 0.27±0.01 | 0.34±0.01 | 0.54±0.01 | 0.52±0.00 |
| **K2T24S10D15** | 0.36±0.00 | 0.32±0.00 | 0.46±0.00 | 0.63±0.00 |  | **K4T28S15D20** | 0.34±0.01 | 0.35±0.01 | 0.54±0.00 | 0.53±0.00 |
| **K2T24S15D15** | 0.35±0.00 | 0.32±0.00 | 0.45±0.00 | 0.77±0.00 |  | **K4T32S10D20** | 0.34±0.01 | 0.33±0.00 | 0.53±0.01 | 0.51±0.00 |
| **K2T28S10D15** | 0.34±0.01 | 0.32±0.00 | 0.53±0.00 | 0.67±0.00 |  | **K4T32S15D20** | 0.34±0.01 | 0.34±0.00 | 0.52±0.01 | 0.52±0.00 |
| **K2T28S15D15** | 0.36±0.01 | 0.32±0.00 | 0.46±0.00 | 0.72±0.00 |  | **K5T24S10D20** | 0.29±0.00 | 0.32±0.00 | 0.46±0.02 | 0.50±0.00 |
| **K2T32S10D15** | 0.36±0.00 | 0.31±0.00 | 0.53±0.00 | 0.70±0.00 |  | **K5T24S15D20** | 0.28±0.00 | 0.35±0.01 | 0.52±0.00 | 0.46±0.00 |
| **K2T32S15D15** | 0.38±0.01 | 0.33±0.00 | 0.52±0.00 | 0.71±0.00 |  | **K5T28S10D20** | 0.28±0.01 | 0.36±0.01 | 0.47±0.01 | 0.43±0.00 |
| **K3T24S10D15** | 0.36±0.00 | 0.32±0.00 | 0.65±0.00 | 0.73±0.01 |  | **K5T28S15D20** | 0.25±0.01 | 0.35±0.01 | 0.52±0.04 | 0.50±0.01 |
| **K3T24S15D15** | 0.36±0.01 | 0.32±0.00 | 0.64±0.02 | 0.72±0.01 |  | **K5T32S10D20** | 0.27±0.00 | 0.33±0.01 | 0.53±0.02 | 0.54±0.00 |
| **K3T28S10D15** | 0.36±0.02 | 0.33±0.00 | 0.54±0.00 | 0.73±0.00 |  | **K5T32S15D20** | 0.29±0.01 | 0.34±0.00 | 0.58±0.01 | 0.50±0.01 |
| **K3T28S15D15** | 0.36±0.01 | 0.33±0.00 | 0.61±0.01 | 0.71±0.00 |  | **K1T24S10D28** | 0.30±0.00 | 0.20±0.01 | 0.41±0.04 | 0.46±0.00 |
| **K3T32S10D15** | 0.35±0.00 | 0.31±0.00 | 0.61±0.00 | 0.71±0.01 |  | **K1T24S15D28** | 0.30±0.01 | 0.19±0.00 | 0.38±0.02 | 0.46±0.00 |
| **K3T32S15D15** | 0.38±0.01 | 0.33±0.00 | 0.61±0.00 | 0.72±0.00 |  | **K1T28S10D28** | 0.38±0.02 | 0.29±0.01 | 0.38±0.00 | 0.44±0.00 |
| **K4T24S10D15** | 0.34±0.01 | 0.30±0.01 | 0.64±0.01 | 0.71±0.00 |  | **K1T28S15D28** | 0.41±0.01 | 0.26±0.01 | 0.37±0.01 | 0.45±0.00 |
| **K4T24S15D15** | 0.34±0.00 | 0.30±0.01 | 0.56±0.01 | 0.72±0.01 |  | **K1T32S10D28** | 0.34±0.00 | 0.29±0.01 | 0.36±0.00 | 0.42±0.00 |
| **K4T28S10D15** | 0.27±0.01 | 0.29±0.00 | 0.49±0.02 | 0.71±0.00 |  | **K1T32S15D28** | 0.35±0.02 | 0.22±0.00 | 0.37±0.00 | 0.44±0.00 |
| **K4T28S15D15** | 0.34±0.01 | 0.31±0.00 | 0.60±0.01 | 0.70±0.00 |  | **K2T24S10D28** | 0.36±0.00 | 0.19±0.00 | 0.42±0.00 | 0.39±0.00 |
| **K4T32S10D15** | 0.34±0.01 | 0.34±0.00 | 0.70±0.00 | 0.70±0.00 |  | **K2T24S15D28** | 0.35±0.00 | 0.20±0.01 | 0.42±0.00 | 0.38±0.00 |
| **K4T32S15D15** | 0.34±0.01 | 0.31±0.00 | 0.68±0.00 | 0.72±0.00 |  | **K2T28S10D28** | 0.34±0.01 | 0.22±0.02 | 0.44±0.00 | 0.41±0.00 |
| **K5T24S10D15** | 0.29±0.00 | 0.33±0.00 | 0.70±0.01 | 0.57±0.01 |  | **K2T28S15D28** | 0.36±0.01 | 0.19±0.01 | 0.41±0.01 | 0.39±0.00 |
| **K5T24S15D15** | 0.28±0.00 | 0.32±0.02 | 0.72±0.01 | 0.70±0.00 |  | **K2T32S10D28** | 0.36±0.00 | 0.22±0.01 | 0.41±0.00 | 0.43±0.00 |
| **K5T28S10D15** | 0.28±0.01 | 0.31±0.00 | 0.63±0.01 | 0.69±0.00 |  | **K2T32S15D28** | 0.38±0.01 | 0.23±0.01 | 0.41±0.01 | 0.39±0.00 |
| **K5T28S15D15** | 0.25±0.01 | 0.31±0.00 | 0.68±0.01 | 0.61±0.01 |  | **K3T24S10D28** | 0.36±0.00 | 0.23±0.00 | 0.41±0.00 | 0.46±0.00 |
| **K5T32S10D15** | 0.27±0.00 | 0.33±0.00 | 0.67±0.01 | 0.69±0.00 |  | **K3T24S15D28** | 0.36±0.01 | 0.22±0.02 | 0.41±0.01 | 0.47±0.00 |
| **K5T32S15D15** | 0.29±0.01 | 0.34±0.00 | 0.62±0.00 | 0.72±0.00 |  | **K3T28S10D28** | 0.36±0.02 | 0.26±0.00 | 0.39±0.00 | 0.44±0.00 |
| **K1T24S10D20** | 0.30±0.00 | 0.37±0.01 | 0.41±0.04 | 0.50±0.00 |  | **K3T28S15D28** | 0.36±0.01 | 0.28±0.02 | 0.39±0.01 | 0.44±0.00 |
| **K1T24S15D20** | 0.30±0.01 | 0.44±0.00 | 0.44±0.01 | 0.49±0.01 |  | **K3T32S10D28** | 0.35±0.00 | 0.26±0.00 | 0.39±0.00 | 0.47±0.00 |
| **K1T28S10D20** | 0.38±0.02 | 0.43±0.00 | 0.40±0.01 | 0.45±0.00 |  | **K3T32S15D28** | 0.38±0.01 | 0.26±0.00 | 0.41±0.00 | 0.45±0.00 |
| **K1T28S15D20** | 0.41±0.01 | 0.41±0.00 | 0.39±0.00 | 0.43±0.00 |  | **K4T24S10D28** | 0.34±0.01 | 0.24±0.02 | 0.37±0.01 | 0.45±0.00 |
| **K1T32S10D20** | 0.34±0.00 | 0.42±0.00 | 0.41±0.00 | 0.49±0.00 |  | **K4T24S15D28** | 0.34±0.00 | 0.25±0.00 | 0.41±0.01 | 0.46±0.00 |
| **K1T32S15D20** | 0.35±0.02 | 0.43±0.01 | 0.39±0.00 | 0.46±0.00 |  | **K4T28S10D28** | 0.27±0.01 | 0.25±0.01 | 0.38±0.00 | 0.45±0.01 |
| **K2T24S10D20** | 0.36±0.00 | 0.30±0.00 | 0.43±0.00 | 0.57±0.01 |  | **K4T28S15D28** | 0.34±0.01 | 0.23±0.01 | 0.40±0.00 | 0.45±0.00 |
| **K2T24S15D20** | 0.35±0.00 | 0.29±0.00 | 0.43±0.00 | 0.54±0.02 |  | **K4T32S10D28** | 0.34±0.01 | 0.22±0.00 | 0.42±0.00 | 0.44±0.00 |
| **K2T28S10D20** | 0.34±0.01 | 0.31±0.00 | 0.50±0.00 | 0.52±0.01 |  | **K4T32S15D28** | 0.34±0.01 | 0.23±0.00 | 0.37±0.01 | 0.46±0.00 |
| **K2T28S15D20** | 0.36±0.01 | 0.30±0.01 | 0.43±0.00 | 0.52±0.00 |  | **K5T24S10D28** | 0.29±0.00 | 0.26±0.00 | 0.50±0.01 | 0.44±0.02 |
| **K2T32S10D20** | 0.36±0.00 | 0.31±0.00 | 0.52±0.00 | 0.54±0.00 |  | **K5T24S15D28** | 0.28±0.00 | 0.26±0.00 | 0.50±0.01 | 0.47±0.00 |
| **K2T32S15D20** | 0.38±0.01 | 0.31±0.00 | 0.45±0.00 | 0.53±0.02 |  | **K5T28S10D28** | 0.28±0.01 | 0.26±0.01 | 0.52±0.02 | 0.46±0.00 |
| **K3T24S10D20** | 0.36±0.00 | 0.36±0.01 | 0.44±0.00 | 0.53±0.00 |  | **K5T28S15D28** | 0.25±0.01 | 0.26±0.00 | 0.50±0.00 | 0.47±0.00 |
| **K3T24S15D20** | 0.36±0.01 | 0.38±0.02 | 0.42±0.01 | 0.55±0.03 |  | **K5T32S10D28** | 0.27±0.00 | 0.21±0.00 | 0.51±0.02 | 0.46±0.00 |
| **K3T28S10D20** | 0.36±0.02 | 0.31±0.00 | 0.49±0.01 | 0.54±0.02 |  | **K5T32S15D28** | 0.29±0.01 | 0.22±0.00 | 0.51±0.01 | 0.47±0.01 |
