# Supplementary material for: Spectral Pattern of Chocolate Production: Early Detection of Quality Problems
Source: J Food Sci. 2026 Jul 20;91(7):e71269. doi: 10.1111/1750-3841.71269 (PMC13383598; doi:10.1111/1750-3841.71269)
Supplement: Supplementary file 1 — Table S1. Hardness changes of chocolate samples within time. Table S2. Water activity changes of chocolate samples. Table S3. Color changes of chocolate samples within time. Table S4. WI changes of chocolate samples within time. Table S5. Moisture, water activity, and particle size values of variously conched samples after conching process. [file JFDS-91-0-s001.zip › jfds71269-sup-0001-TableS3.docx]

| Sample | Color (L*) | | | | Color (a*) | | | | Color (b*) | | | |
| --- | --- | --- | --- | --- | --- | --- | --- | --- | --- | --- | --- | --- |
|  | t=0 | t=2 | t=4 | t=8 | t=0 | t=2 | t=4 | t=8 | t=0 | t=2 | t=4 | t=8 |
| **K1T24S10D15** | 39.59±0.03 | 37.69±1.96 | 39.30±0.12 | 38.22±0.48 | 10.36±0.02 | 10.52±0.06 | 10.30±0.13 | 9.98±0.22 | 14.69±0.03 | 14.63±0.31 | 14.41±0.17 | 13.07±0.33 |
| **K1T24S15D15** | 40.71±0.17 | 38.61±0.46 | 39.16±0.06 | 38.05±0.15 | 10.24±0.07 | 10.41±0.10 | 10.20±0.05 | 10.12±0.02 | 14.5±0.10 | 14.43±0.18 | 14.23±0.06 | 13.43±0.08 |
| **K1T28S10D15** | 38.16±0.05 | 37.84±0.01 | 38.03±0.20 | 36.77±0.18 | 10.25±0.01 | 10.32±0.04 | 10.17±0.06 | 9.94±0.10 | 13.88±0.02 | 13.80±0.10 | 13.66±0.11 | 12.51±0.32 |
| **K1T28S15D15** | 39.54±0.16 | 37.84±0.85 | 38.44±0.14 | 37.15±0.36 | 10.16±0.01 | 10.24±0.08 | 10.11±0.11 | 9.97±0.02 | 13.84±0.02 | 13.94±0.11 | 13.88±0.12 | 12.77±0.23 |
| **K1T32S10D15** | 38.37±0.13 | 38.31±0.22 | 38.71±0.28 | 37.86±0.35 | 10.13±0.02 | 10.21±0.04 | 10.10±0.05 | 10.01±0.07 | 13.9±0.09 | 13.97±0.06 | 14.05±0.11 | 13.33±0.26 |
| **K1T32S15D15** | 39.55±0.07 | 38.87±0.62 | 38.61±0.11 | 36.60±0.16 | 10.11±0.04 | 10.31±0.07 | 10.01±0.03 | 9.89±0.05 | 13.79±0.09 | 14.37±0.27 | 13.78±0.09 | 12.29±0.17 |
| **K2T24S10D15** | 39.10±0.12 | 38.45±0.24 | 38.39±0.27 | 37.84±0.51 | 10.00±0.07 | 10.15±0.03 | 9.99±0.05 | 9.88±0.02 | 13.95±0.05 | 13.87±0.12 | 13.56±0.14 | 12.98±0.15 |
| **K2T24S15D15** | 38.24±0.26 | 37.45±0.13 | 37.45±0.08 | 36.70±0.22 | 9.85±0.05 | 9.96±0.02 | 9.86±0.07 | 9.63±0.05 | 13.46±0.06 | 13.21±0.03 | 13.08±0.14 | 12.25±0.08 |
| **K2T28S10D15** | 37.45±0.06 | 36.96±0.19 | 37.16±0.30 | 36.13±0.25 | 9.72±0.09 | 9.83±0.05 | 9.65±0.07 | 9.54±0.02 | 13.12±0.19 | 12.89±0.10 | 12.68±0.15 | 11.90±0.15 |
| **K2T28S15D15** | 37.66±0.17 | 37.20±0.21 | 37.39±0.28 | 36.65±0.15 | 9.83±0.11 | 9.71±0.02 | 9.56±0.07 | 9.52±0.05 | 13.36±0.22 | 12.91±0.15 | 12.77±0.10 | 12.20±0.04 |
| **K2T32S10D15** | 37.71±0.06 | 37.03±0.06 | 37.21±0.08 | 36.24±0.20 | 9.61±0.05 | 9.76±0.07 | 9.65±0.06 | 9.53±0.03 | 13.00±0.04 | 12.82±0.07 | 12.74±0.10 | 11.97±0.12 |
| **K2T32S15D15** | 37.76±0.06 | 37.76±0.19 | 37.94±0.33 | 37.20±0.31 | 9.77±0.06 | 9.70±0.09 | 9.63±0.13 | 9.60±0.17 | 13.28±0.16 | 13.04±0.09 | 13.19±0.29 | 12.62±0.40 |
| **K3T24S10D15** | 38.57±9.92 | 37.95±0.07 | 38.09±0.16 | 37.43±0.39 | 9.92±0.02 | 10.15±0.06 | 10.01±0.06 | 9.71±0.08 | 13.68±0.12 | 13.64±0.21 | 13.55±0.18 | 12.56±0.16 |
| **K3T24S15D15** | 39.06±10.12 | 39.33±10.09 | 39.80±0.44 | 38.84±0.40 | 10.12±0.09 | 10.09±0.07 | 10.02±0.04 | 10.04±0.08 | 14.05±0.06 | 13.97±0.03 | 14.16±0.21 | 13.77±0.18 |
| **K3T28S10D15** | 37.27±9.75 | 37.05±9.87 | 37.41±0.12 | 36.04±0.06 | 9.75±0.04 | 9.87±0.04 | 9.65±0.04 | 9.47±0.08 | 12.98±0.09 | 12.92±0.13 | 12.81±0.21 | 11.79±0.15 |
| **K3T28S15D15** | 37.09±9.74 | 37.14±9.83 | 37.69±0.24 | 36.54±0.02 | 9.74±0.02 | 9.83±0.12 | 9.63±0.05 | 9.46±0.01 | 13.02±0.07 | 12.97±0.37 | 12.96±0.09 | 12.08±0.02 |
| **K3T32S10D15** | 37.22±9.69 | 36.81±9.82 | 37.44±0.04 | 36.27±0.02 | 9.69±0.03 | 9.82±0.01 | 9.65±0.03 | 9.45±0.02 | 12.86±0.02 | 12.72±0.03 | 12.77±0.07 | 11.80±0.03 |
| **K3T32S15D15** | 37.23±9.97 | 37.43±9.99 | 38.34±0.24 | 36.54±0.19 | 9.97±0.04 | 9.99±0.04 | 9.70±0.18 | 9.66±0.09 | 13.21±0.08 | 13.15±0.09 | 13.35±0.21 | 12.10±0.06 |
| **K4T24S10D15** | 38.16±0.06 | 37.73±0.06 | 37.59±0.08 | 38.55±0.04 | 9.87±0.04 | 10.06±0.05 | 10.12±0.01 | 8.44±0.05 | 13.45±0.15 | 13.40±0.09 | 13.33±0.04 | 9.48±0.05 |
| **K4T24S15D15** | 39.12±0.34 | 39.51±1.24 | 39.46±0.64 | 40.84±0.25 | 10.21±0.04 | 10.25±0.24 | 10.24±0.02 | 8.95±0.02 | 14.33±0.17 | 14.29±0.75 | 14.22±0.32 | 10.98±0.12 |
| **K4T28S10D15** | 37.96±0.13 | 38.23±0.12 | 38.19±0.30 | 39.25±0.11 | 9.86±0.08 | 9.92±0.03 | 9.98±0.06 | 8.52±0.02 | 13.44±0.17 | 13.33±0.04 | 13.27±0.06 | 9.89±0.10 |
| **K4T28S15D15** | 37.72±0.21 | 37.90±0.09 | 38.03±0.10 | 38.57±0.10 | 9.97±0.04 | 9.96±0.02 | 9.95±0.04 | 8.46±0.06 | 13.54±0.12 | 13.41±0.05 | 13.35±0.06 | 9.47±0.06 |
| **K4T32S10D15** | 37.78±0.05 | 37.71±0.22 | 37.93±0.09 | 38.58±0.05 | 9.83±0.02 | 9.89±0.04 | 9.89±0.05 | 8.47±0.03 | 13.39±0.07 | 13.16±0.11 | 13.15±0.11 | 9.61±0.07 |
| **K4T32S15D15** | 38.30±0.25 | 38.10±0.05 | 38.54±0.44 | 38.64±0.10 | 9.97±0.02 | 9.92±0.01 | 9.90±0.07 | 8.47±0.04 | 13.67±0.08 | 13.30±0.05 | 13.40±0.10 | 9.69±0.02 |
| **K5T24S10D15** | 38.47±0.03 | 38.34±0.12 | 38.66±0.08 | 39.16±0.28 | 9.41±0.05 | 9.61±0.02 | 9.66±0.01 | 8.11±0.07 | 13.24±0.14 | 13.32±0.10 | 13.38±0.02 | 9.55±0.28 |
| **K5T24S15D15** | 39.27±0.06 | 38.51±0.03 | 38.65±0.19 | 39.43±0.24 | 9.95±0.03 | 10.14±0.11 | 10.20±0.05 | 8.79±0.06 | 13.95±0.06 | 13.89±0.20 | 13.96±0.16 | 10.27±0.17 |
| **K5T28S10D15** | 38.29±0.14 | 37.75±0.26 | 37.93±0.11 | 38.55±0.16 | 9.66±0.05 | 9.78±0.05 | 9.91±0.04 | 8.41±0.09 | 13.33±0.08 | 13.14±0.11 | 13.28±0.13 | 9.67±0.16 |
| **K5T28S15D15** | 38.76±0.37 | 38.04±0.17 | 38.25±0.22 | 38.77±0.28 | 9.90±0.08 | 9.93±0.02 | 10.01±0.04 | 8.52±0.05 | 13.65±0.09 | 13.36±0.03 | 13.47±0.04 | 9.73±0.07 |
| **K5T32S10D15** | 38.03±0.17 | 37.65±0.16 | 38.06±0.15 | 38.81±0.13 | 9.76±0.03 | 9.88±0.07 | 9.93±0.06 | 8.37±0.06 | 13.28±0.05 | 13.18±0.13 | 13.28±0.06 | 9.56±0.07 |
| **K5T32S15D15** | 38.40±0.06 | 38.00±0.10 | 38.50±0.34 | 38.59±0.12 | 9.87±0.04 | 9.89±0.03 | 9.97±0.06 | 8.37±0.21 | 13.47±0.04 | 13.27±0.04 | 13.45±0.08 | 9.43±0.33 |
| **K1T24S10D20** | 39.59±0.03 | 39.20±0.06 | 39.75±0.05 | 39.33±0.12 | 10.36±0.02 | 10.32±0.04 | 10.15±0.01 | 10.26±0.08 | 14.69±0.03 | 14.26±0.09 | 14.27±0.05 | 14.24±0.20 |
| **K1T24S15D20** | 39.57±0.33 | 38.83±0.14 | 39.10±0.64 | 39.04±0.26 | 10.24±0.07 | 10.35±0.08 | 10.11±0.07 | 10.17±0.05 | 14.5±0.10 | 14.29±0.17 | 14.28±0.15 | 13.98±0.14 |
| **K1T28S10D20** | 38.16±0.05 | 37.70±0.15 | 38.27±0.14 | 38.11±0.51 | 10.25±0.01 | 10.25±0.06 | 10.03±0.04 | 10.11±0.18 | 13.88±0.02 | 13.70±0.11 | 13.67±0.07 | 13.61±0.06 |
| **K1T28S15D20** | 38.25±0.11 | 38.22±0.17 | 38.65±0.02 | 38.22±0.14 | 10.16±0.01 | 10.18±0.03 | 9.92±0.01 | 10.05±0.03 | 13.84±0.02 | 13.78±0.12 | 13.64±0.06 | 13.57±0.07 |
| **K1T32S10D20** | 38.37±0.13 | 38.33±0.29 | 38.33±0.66 | 38.54±0.24 | 10.13±0.02 | 10.13±0.11 | 9.84±0.08 | 9.96±0.12 | 13.9±0.09 | 13.71±0.12 | 13.70±0.29 | 13.49±0.18 |
| **K1T32S15D20** | 38.35±0.16 | 38.38±0.30 | 38.92±0.13 | 38.37±0.20 | 10.11±0.04 | 10.03±0.06 | 9.83±0.09 | 9.96±0.09 | 13.79±0.09 | 13.57±0.08 | 13.53±0.10 | 13.47±0.21 |
| **K2T24S10D20** | 39.10±0.12 | 37.97±0.32 | 38.75±0.11 | 38.64±0.40 | 10.00±0.07 | 10.10±0.03 | 9.87±0.20 | 9.88±0.07 | 13.95±0.05 | 13.49±0.13 | 13.55±0.31 | 13.37±0.14 |
| **K2T24S15D20** | 38.24±0.26 | 37.55±0.25 | 38.27±0.24 | 38.29±0.18 | 9.85±0.05 | 9.85±0.04 | 9.66±0.02 | 9.68±0.00 | 13.46±0.06 | 13.04±0.03 | 13.15±0.12 | 13.09±0.07 |
| **K2T28S10D20** | 37.45±0.06 | 36.63±0.15 | 37.57±0.15 | 37.52±0.16 | 9.72±0.09 | 9.66±0.10 | 9.52±0.05 | 9.46±0.05 | 13.12±0.19 | 12.43±0.15 | 12.75±0.14 | 12.42±0.06 |
| **K2T28S15D20** | 37.66±0.17 | 37.05±0.10 | 37.82±0.20 | 37.72±0.20 | 9.83±0.11 | 9.75±0.02 | 9.57±0.09 | 9.56±0.07 | 13.36±0.22 | 12.78±0.07 | 12.88±0.19 | 12.68±0.11 |
| **K2T32S10D20** | 37.71±0.06 | 36.84±0.12 | 37.79±0.20 | 37.89±0.11 | 9.61±0.05 | 9.63±0.03 | 9.45±0.07 | 9.43±0.11 | 13.00±0.04 | 12.52±0.03 | 12.72±0.13 | 12.60±0.19 |
| **K2T32S15D20** | 37.76±0.06 | 37.53±0.06 | 38.26±0.10 | 37.91±0.17 | 9.77±0.06 | 9.60±0.07 | 9.43±0.07 | 9.52±0.03 | 13.28±0.16 | 12.75±0.15 | 12.99±0.19 | 12.76±0.15 |
| **K3T24S10D20** | 38.57±9.92 | 38.06±9.89 | 38.62±9.69 | 38.54±0.14 | 9.92±0.02 | 9.84±0.05 | 9.69±0.12 | 9.84±0.04 | 13.68±0.12 | 13.21±0.12 | 13.21±0.13 | 13.35±0.16 |
| **K3T24S15D20** | 39.06±10.12 | 39.11±10.03 | 39.83±9.94 | 39.29±0.01 | 10.12±0.09 | 10.03±0.07 | 9.94±0.06 | 10.08±0.08 | 14.05±0.06 | 13.86±0.16 | 14.14±0.21 | 14.09±0.15 |
| **K3T28S10D20** | 37.27±9.75 | 37.25±9.75 | 37.48±9.62 | 37.43±0.16 | 9.75±0.04 | 9.75±0.06 | 9.62±0.03 | 9.62±0.01 | 12.98±0.09 | 12.74±0.12 | 12.79±0.06 | 12.64±0.10 |
| **K3T28S15D20** | 37.09±9.74 | 37.42±9.56 | 37.77±9.46 | 37.51±0.22 | 9.74±0.02 | 9.56±0.06 | 9.46±0.03 | 9.56±0.13 | 13.02±0.07 | 12.70±0.11 | 12.85±0.04 | 12.74±0.15 |
| **K3T32S10D20** | 37.22±9.69 | 37.33±9.67 | 37.72±9.43 | 37.31±0.31 | 9.69±0.03 | 9.67±0.08 | 9.43±0.01 | 9.58±0.05 | 12.86±0.02 | 12.79±0.17 | 12.7±0.10 | 12.65±0.08 |
| **K3T32S15D20** | 37.23±9.97 | 38.04±9.87 | 38.51±9.77 | 38.05±0.18 | 9.97±0.04 | 9.87±0.10 | 9.77±0.04 | 9.85±0.14 | 13.21±0.08 | 13.33±0.10 | 13.51±0.16 | 13.30±0.23 |
| **K4T24S10D20** | 38.16±0.06 | 37.91±0.05 | 39.57±0.13 | 40.12±0.16 | 9.87±0.04 | 9.95±0.03 | 10.25±0.02 | 8.92±0.04 | 13.45±0.15 | 13.25±0.06 | 14.32±0.07 | 10.96±0.01 |
| **K4T24S15D20** | 39.12±0.34 | 39.62±0.67 | 38.02±0.03 | 41.43±0.50 | 10.21±0.04 | 10.05±0.08 | 9.95±0.03 | 9.18±0.11 | 14.33±0.17 | 13.97±0.17 | 13.15±0.09 | 11.74±0.19 |
| **K4T28S10D20** | 37.96±0.13 | 37.91±0.05 | 38.06±0.21 | 40.10±0.10 | 9.86±0.08 | 9.91±0.05 | 9.83±0.08 | 8.84±0.03 | 13.44±0.17 | 13.22±0.07 | 13.04±0.06 | 10.85±0.04 |
| **K4T28S15D20** | 37.72±0.21 | 37.75±0.25 | 37.85±0.20 | 40.04±0.24 | 9.97±0.04 | 9.94±0.03 | 9.89±0.03 | 8.87±0.05 | 13.54±0.12 | 13.22±0.07 | 13.17±0.11 | 10.93±0.19 |
| **K4T32S10D20** | 37.78±0.05 | 37.62±0.11 | 37.86±0.05 | 39.70±0.09 | 9.83±0.02 | 9.87±0.02 | 9.82±0.02 | 8.76±0.09 | 13.39±0.07 | 13.14±0.01 | 13.06±0.03 | 10.65±0.05 |
| **K4T32S15D20** | 38.30±0.25 | 37.95±0.10 | 37.97±0.07 | 40.18±0.25 | 9.97±0.02 | 9.91±0.02 | 9.93±0.03 | 8.76±0.08 | 13.67±0.08 | 13.19±0.01 | 13.17±0.14 | 10.71±0.04 |
| **K5T24S10D20** | 38.47±0.03 | 37.75±0.02 | 37.90±0.15 | 40.14±0.28 | 9.41±0.05 | 9.55±0.03 | 9.47±0.08 | 8.56±0.08 | 13.24±0.14 | 12.96±0.10 | 12.76±0.04 | 10.67±0.11 |
| **K5T24S15D20** | 39.27±0.06 | 38.42±0.05 | 38.64±0.35 | 40.98±0.24 | 9.95±0.03 | 10.05±0.03 | 10.02±0.07 | 8.91±0.12 | 13.95±0.06 | 13.58±0.04 | 13.55±0.05 | 11.19±0.16 |
| **K5T28S10D20** | 38.29±0.14 | 37.55±0.14 | 37.49±0.27 | 39.78±0.03 | 9.66±0.05 | 9.79±0.05 | 9.75±0.06 | 8.75±0.03 | 13.33±0.08 | 13.01±0.12 | 12.93±0.15 | 10.80±0.01 |
| **K5T28S15D20** | 38.76±0.37 | 38.08±0.29 | 38.20±0.34 | 40.14±0.07 | 9.90±0.08 | 9.95±0.08 | 9.89±0.08 | 8.86±0.06 | 13.65±0.09 | 13.26±0.08 | 13.40±0.35 | 10.82±0.08 |
| **K5T32S10D20** | 38.03±0.17 | 37.43±0.12 | 37.62±0.18 | 40.79±0.78 | 9.76±0.03 | 9.84±0.02 | 9.81±0.09 | 8.50±0.22 | 13.28±0.05 | 12.99±0.07 | 12.83±0.09 | 10.66±0.18 |
| **K5T32S15D20** | 38.40±0.06 | 37.74±0.08 | 37.76±0.03 | 39.83±0.20 | 9.87±0.04 | 9.85±0.02 | 9.84±0.03 | 8.81±0.07 | 13.47±0.04 | 13.05±0.05 | 12.94±0.02 | 10.66±0.03 |
| **K1T24S10D28** | 39.59±0.03 | 40.55±0.34 | 42.61±0.10 | 43.12±0.18 | 10.36±0.02 | 10.17±0.13 | 10.22±0.03 | 10.21±0.09 | 14.69±0.03 | 14.54±0.13 | 15.49±0.05 | 15.55±0.05 |
| **K1T24S15D28** | 40.71±0.17 | 39.95±0.36 | 42.23±0.32 | 42.65±0.20 | 10.24±0.07 | 10.33±0.08 | 10.16±0.02 | 10.14±0.02 | 14.50±0.10 | 14.74±0.09 | 15.30±0.11 | 15.28±0.13 |
| **K1T28S10D28** | 38.16±0.05 | 38.81±0.64 | 43.16±1.08 | 43.14±0.23 | 10.25±0.01 | 10.26±0.04 | 10.12±0.35 | 10.21±0.08 | 13.88±0.02 | 14.03±0.07 | 15.51±0.67 | 15.51±0.03 |
| **K1T28S15D28** | 39.54±0.16 | 38.82±0.21 | 42.01±0.45 | 42.78±0.56 | 10.16±0.01 | 10.21±0.09 | 10.16±0.08 | 9.94±0.05 | 13.84±0.02 | 14.09±0.04 | 15.17±0.18 | 14.86±0.05 |
| **K1T32S10D28** | 38.37±0.13 | 39.38±0.41 | 42.38±0.25 | 43.90±1.71 | 10.13±0.02 | 10.23±0.03 | 10.13±0.07 | 9.18±0.56 | 13.90±0.09 | 13.97±0.12 | 15.26±0.15 | 14.48±0.30 |
| **K1T32S15D28** | 39.55±0.07 | 38.99±0.11 | 41.41±0.08 | 43.10±0.84 | 10.11±0.04 | 10.2±0.09 | 9.96±0.09 | 9.52±0.27 | 13.79±0.09 | 14.09±0.03 | 14.62±0.18 | 14.41±0.22 |
| **K2T24S10D28** | 39.10±0.12 | 41.62±0.32 | 42.20±0.23 | 42.24±0.20 | 10.00±0.07 | 10.00±0.06 | 9.91±0.04 | 9.91±0.05 | 13.95±0.05 | 14.99±0.27 | 14.99±0.16 | 14.92±0.14 |
| **K2T24S15D28** | 38.24±0.26 | 40.63±0.35 | 41.14±0.21 | 41.85±0.37 | 9.85±0.05 | 9.83±0.03 | 9.78±0.07 | 9.74±0.04 | 13.46±0.06 | 14.29±0.11 | 14.39±0.12 | 14.49±0.16 |
| **K2T28S10D28** | 37.45±0.06 | 39.95±0.12 | 40.74±0.19 | 42.63±1.10 | 9.72±0.09 | 9.68±0.01 | 9.62±0.05 | 9.29±0.32 | 13.12±0.19 | 13.77±0.09 | 13.85±0.13 | 13.87±0.17 |
| **K2T28S15D28** | 37.66±0.17 | 40.34±0.11 | 41.07±0.17 | 46.73±0.81 | 9.83±0.11 | 9.65±0.03 | 9.71±0.09 | 8.23±0.17 | 13.36±0.22 | 13.89±0.02 | 14.27±0.34 | 13.77±0.32 |
| **K2T32S10D28** | 37.71±0.06 | 39.83±0.35 | 40.96±0.07 | 45.19±0.63 | 9.61±0.05 | 9.62±0.01 | 9.71±0.08 | 8.45±0.25 | 13.00±0.04 | 12.27±2.41 | 14.20±0.19 | 13.42±0.18 |
| **K2T32S15D28** | 37.76±0.06 | 40.02±0.28 | 40.57±0.43 | 50.91±4.68 | 9.77±0.06 | 9.55±0.09 | 9.51±0.14 | 7.18±1.23 | 13.28±0.16 | 13.62±0.12 | 13.74±0.26 | 13.80±0.39 |
| **K3T24S10D28** | 38.57±9.92 | 41.64±9.98 | 41.07±9.69 | 42.23±0.39 | 9.92±0.02 | 9.98±0.08 | 9.69±0.12 | 9.73±0.17 | 13.68±0.12 | 14.86±0.25 | 14.38±0.05 | 14.65±0.32 |
| **K3T24S15D28** | 39.06±10.12 | 41.14±9.93 | 41.31±9.82 | 41.93±0.30 | 10.12±0.09 | 9.93±0.08 | 9.82±0.01 | 9.76±0.04 | 14.05±0.06 | 14.5±0.18 | 14.41±0.08 | 14.49±0.20 |
| **K3T28S10D28** | 37.27±9.75 | 40.54±9.93 | 40.90±9.70 | 45.62±1.42 | 9.75±0.04 | 9.93±0.11 | 9.70±0.09 | 7.71±0.51 | 12.98±0.09 | 14.54±0.44 | 14.22±0.25 | 12.96±0.85 |
| **K3T28S15D28** | 37.09±9.74 | 40.09±9.74 | 40.24±9.57 | 45.53±0.65 | 9.74±0.02 | 9.74±0.06 | 9.57±0.06 | 8.13±0.13 | 13.02±0.07 | 13.84±0.10 | 13.78±0.13 | 13.58±0.09 |
| **K3T32S10D28** | 37.22±9.69 | 40.06±9.78 | 40.78±9.53 | 44.75±1.22 | 9.69±0.03 | 9.78±0.09 | 9.53±0.13 | 8.68±0.21 | 12.86±0.02 | 13.99±0.27 | 13.87±0.31 | 13.86±0.22 |
| **K3T32S15D28** | 37.23±9.97 | 39.62±9.77 | 40.33±9.51 | 40.99±0.08 | 9.97±0.04 | 9.77±0.05 | 9.51±0.08 | 9.73±0.06 | 13.21±0.08 | 13.67±0.22 | 13.44±0.06 | 14.00±0.05 |
| **K4T24S10D28** | 38.16±0.06 | 41.73±0.26 | 42.56±0.26 | 44.40±0.42 | 9.87±0.04 | 9.85±0.07 | 9.91±0.06 | 8.91±0.29 | 13.45±0.15 | 14.59±0.09 | 14.99±0.28 | 12.71±0.17 |
| **K4T24S15D28** | 39.12±0.34 | 42.28±0.15 | 43.10±0.26 | 44.71±0.31 | 10.21±0.04 | 10.14±0.14 | 10.19±0.06 | 9.24±0.13 | 14.33±0.17 | 15.24±0.22 | 15.55±0.06 | 13.21±0.28 |
| **K4T28S10D28** | 37.96±0.13 | 40.69±0.12 | 41.87±0.30 | 45.72±0.45 | 9.86±0.08 | 9.88±0.09 | 9.93±0.05 | 8.53±0.06 | 13.44±0.17 | 14.31±0.22 | 14.74±0.13 | 12.47±0.10 |
| **K4T28S15D28** | 37.72±0.21 | 41.16±0.22 | 42.11±0.31 | 46.28±1.31 | 9.97±0.04 | 9.83±0.02 | 9.94±0.07 | 8.11±0.37 | 13.54±0.12 | 14.38±0.07 | 14.92±0.16 | 11.84±0.32 |
| **K4T32S10D28** | 37.78±0.05 | 40.51±0.29 | 41.80±0.18 | 50.68±3.50 | 9.83±0.02 | 9.81±0.05 | 9.93±0.02 | 7.25±0.86 | 13.39±0.07 | 14.09±0.12 | 14.64±0.09 | 12.42±0.38 |
| **K4T32S15D28** | 38.30±0.25 | 40.38±0.33 | 41.49±0.07 | 61.38±3.29 | 9.97±0.02 | 9.70±0.22 | 9.91±0.01 | 5.91±0.51 | 13.67±0.08 | 13.94±0.33 | 14.56±0.08 | 13.96±0.48 |
| **K5T24S10D28** | 38.47±0.03 | 40.67±0.46 | 41.35±0.09 | 43.44±0.31 | 9.41±0.05 | 9.36±0.16 | 9.54±0.03 | 8.84±0.06 | 13.24±0.14 | 13.84±0.27 | 14.24±0.03 | 12.40±0.08 |
| **K5T24S15D28** | 39.27±0.06 | 40.99±0.22 | 41.64±0.13 | 46.00±2.13 | 9.95±0.03 | 9.82±0.16 | 9.89±0.08 | 8.77±0.99 | 13.95±0.06 | 14.22±0.19 | 14.50±0.11 | 12.69±1.05 |
| **K5T28S10D28** | 38.29±0.14 | 41.59±1.18 | 41.60±0.25 | 43.79±0.46 | 9.66±0.05 | 9.30±0.34 | 9.64±0.12 | 8.76±0.14 | 13.33±0.08 | 13.52±0.52 | 14.20±0.23 | 12.10±0.31 |
| **K5T28S15D28** | 38.76±0.37 | 40.81±0.60 | 41.51±0.22 | 43.60±0.34 | 9.90±0.08 | 9.69±0.15 | 9.82±0.04 | 9.07±0.04 | 13.65±0.09 | 14.00±0.21 | 14.45±0.06 | 12.52±0.22 |
| **K5T32S10D28** | 38.03±0.17 | 40.07±0.28 | 41.01±0.10 | 60.14±1.81 | 9.76±0.03 | 9.62±0.16 | 9.70±0.09 | 6.35±0.19 | 13.28±0.05 | 13.66±0.20 | 14.04±0.10 | 15.09±0.39 |
| **K5T32S15D28** | 38.40±0.06 | 40.25±0.11 | 40.88±0.22 | 60.99±2.79 | 9.87±0.04 | 9.75±0.08 | 9.78±0.11 | 5.75±0.40 | 13.47±0.04 | 13.90±0.10 | 14.14±0.20 | 13.41±0.37 |
