# Supplementary material for: Spectral Pattern of Chocolate Production: Early Detection of Quality Problems
Source: J Food Sci. 2026 Jul 20;91(7):e71269. doi: 10.1111/1750-3841.71269 (PMC13383598; doi:10.1111/1750-3841.71269)
Supplement: Supplementary file 1 — Table S1. Hardness changes of chocolate samples within time. Table S2. Water activity changes of chocolate samples. Table S3. Color changes of chocolate samples within time. Table S4. WI changes of chocolate samples within time. Table S5. Moisture, water activity, and particle size values of variously conched samples after conching process. [file JFDS-91-0-s001.zip › jfds71269-sup-0001-TableS4.docx]

| Sample | Whiteness Index | | | |  | Sample | Whiteness Index | | | |
| --- | --- | --- | --- | --- | --- | --- | --- | --- | --- | --- |
|  | t=0 | t=2 | t=4 | t=8 |  |  | t=0 | t=2 | t=4 | t=8 |
| **K1T24S10D15** | 36.98±0.03 | 34.88±1.96 | 36.77±0.18 | 36.07±0.54 |  | **K3T28S15D20** | 35.02±0.08 | 38.58±0.24 | 35.63±0.25 | 35.51±0.26 |
| **K1T24S15D15** | 35.80±0.05 | 36.88±0.50 | 35.73±0.18 | 35.81±0.13 |  | **K3T32S10D20** | 35.19±0.07 | 37.75±0.07 | 35.73±0.16 | 35.33±0.32 |
| **K1T28S10D15** | 36.02±0.10 | 36.28±0.02 | 36.31±0.26 | 34.78±0.10 |  | **K3T32S15D20** | 35.08±0.45 | 37.32±0.05 | 36.09±0.12 | 35.88±0.21 |
| **K1T28S15D15** | 37.01±0.31 | 35.79±0.80 | 36.69±0.08 | 35.10±0.31 |  | **K4T24S10D20** | 35.95±0.08 | 39.13±0.25 | 39.81±0.17 | 38.48±0.16 |
| **K1T32S10D15** | 35.91±0.10 | 36.71±0.20 | 36.09±0.18 | 35.66±0.28 |  | **K4T24S15D20** | 36.63±0.29 | 39.44±0.11 | 40.14±0.26 | 39.56±0.47 |
| **K1T32S15D15** | 36.02±0.13 | 36.82±0.54 | 36.29±0.09 | 34.67±0.12 |  | **K4T28S10D20** | 35.76±0.10 | 38.20±0.11 | 39.22±0.33 | 38.49±0.09 |
| **K2T24S10D15** | 36.73±0.14 | 36.09±0.21 | 36.13±0.22 | 35.74±0.46 |  | **K4T28S15D20** | 35.49±0.18 | 38.64±0.19 | 39.40±0.24 | 38.41±0.19 |
| **K2T24S15D15** | 35.35±0.11 | 35.30±0.11 | 35.17±0.27 | 34.72±0.16 |  | **K4T32S10D20** | 35.60±0.05 | 38.08±0.25 | 39.17±0.16 | 38.14±0.08 |
| **K2T28S10D15** | 35.64±0.05 | 34.91±0.17 | 35.21±0.09 | 34.39±0.27 |  | **K4T32S15D20** | 36.02±0.22 | 38.01±0.22 | 38.90±0.06 | 38.60±0.25 |
| **K2T28S15D15** | 36.02±0.27 | 35.16±0.17 | 35.34±0.04 | 34.85±0.14 |  | **K5T24S10D20** | 36.37±0.04 | 35.70±0.04 | 35.90±0.16 | 38.60±0.24 |
| **K2T32S10D15** | 35.49±0.21 | 35.01±0.08 | 35.39±0.26 | 34.46±0.23 |  | **K5T24S15D20** | 36.90±0.05 | 35.46±0.10 | 36.37±0.34 | 39.27±0.26 |
| **K2T32S15D15** | 35.61±0.02 | 35.67±0.21 | 35.82±0.32 | 35.3±0.40 |  | **K5T28S10D20** | 36.14±0.12 | 35.34±0.11 | 35.43±0.29 | 38.20±0.03 |
| **K3T24S10D15** | 36.29±0.15 | 35.67±0.04 | 35.61±0.08 | 35.45±0.36 |  | **K5T28S15D20** | 36.48±0.39 | 36.14±0.06 | 36.00±0.29 | 38.52±0.05 |
| **K3T24S15D15** | 36.64±0.33 | 34.98±0.06 | 37.03±0.27 | 36.51±0.38 |  | **K5T32S10D20** | 35.87±0.16 | 35.90±0.31 | 35.56±0.20 | 39.24±0.79 |
| **K3T28S10D15** | 35.20±0.11 | 34.80±0.12 | 34.98±0.05 | 34.28±0.03 |  | **K5T32S15D20** | 36.17±0.06 | 35.63±0.09 | 35.68±0.03 | 38.26±0.20 |
| **K3T28S15D15** | 35.02±0.08 | 36.93±0.3 | 35.11±0.09 | 34.71±0.02 |  | **K1T24S10D28** | 36.98±0.03 | 37.53±0.04 | 39.69±0.11 | 40.15±0.19 |
| **K3T32S10D15** | 35.19±0.07 | 35.07±0.24 | 35.11±0.10 | 34.50±0.01 |  | **K1T24S15D28** | 35.80±0.05 | 37.13±0.16 | 40.22±0.86 | 39.79±0.15 |
| **K3T32S15D15** | 35.08±0.45 | 35.29±0.16 | 35.58±0.29 | 34.68±0.19 |  | **K1T28S10D28** | 36.02±0.10 | 36.10±0.17 | 39.54±0.23 | 40.19±0.24 |
| **K4T24S10D15** | 35.95±0.08 | 35.52±0.08 | 35.38±0.07 | 37.25±0.04 |  | **K1T28S15D28** | 37.01±0.31 | 36.73±0.15 | 39.38±0.28 | 40.05±0.55 |
| **K4T24S15D15** | 36.63±0.29 | 37.00±0.19 | 36.97±0.54 | 39.17±0.23 |  | **K1T32S10D28** | 35.91±0.10 | 36.91±0.32 | 39.20±0.44 | 41.34±1.60 |
| **K4T28S10D15** | 35.76±0.10 | 36.04±0.11 | 36.00±0.29 | 37.86±0.09 |  | **K1T32S15D28** | 36.02±0.13 | 37.04±0.29 | 38.80±0.02 | 40.54±0.88 |
| **K4T28S15D15** | 35.49±0.18 | 35.69±0.07 | 35.83±0.09 | 37.27±0.08 |  | **K2T24S10D28** | 36.73±0.14 | 38.90±0.23 | 39.47±0.18 | 39.54±0.25 |
| **K4T32S10D15** | 35.60±0.05 | 35.57±0.19 | 35.78±0.06 | 37.26±0.04 |  | **K2T24S15D28** | 35.35±0.11 | 38.15±0.31 | 38.39±0.15 | 39.34±0.42 |
| **K4T32S15D15** | 36.02±0.22 | 35.91±0.04 | 36.32±0.42 | 37.31±0.10 |  | **K2T28S10D28** | 35.64±0.05 | 37.64±0.12 | 38.50±0.05 | 40.85±0.69 |
| **K5T24S10D15** | 36.37±0.04 | 36.19±0.09 | 36.47±0.08 | 37.88±0.23 |  | **K2T28S15D28** | 36.02±0.27 | 37.99±0.12 | 38.62±0.16 | 43.92±0.13 |
| **K5T24S15D15** | 36.90±0.05 | 35.63±0.24 | 36.26±0.16 | 37.94±0.20 |  | **K2T32S10D28** | 35.49±0.21 | 37.54±0.32 | 38.60±0.09 | 43.27±0.38 |
| **K5T28S10D15** | 36.14±0.12 | 35.51±0.19 | 35.76±0.10 | 37.23±0.18 |  | **K2T32S15D28** | 35.61±0.02 | 37.75±0.26 | 38.26±0.35 | 46.54±4.69 |
| **K5T28S15D15** | 36.48±0.39 | 36.15±0.04 | 36.01±0.20 | 37.42±0.28 |  | **K3T24S10D28** | 36.29±0.15 | 35.90±0.14 | 39.28±0.21 | 39.62±0.38 |
| **K5T32S10D15** | 35.87±0.16 | 35.85±0.16 | 35.88±0.16 | 37.51±0.14 |  | **K3T24S15D28** | 36.64±0.33 | 35.23±0.06 | 38.74±0.27 | 39.36±0.25 |
| **K5T32S15D15** | 36.17±0.06 | 35.83±0.10 | 36.26±0.32 | 37.31±0.19 |  | **K3T28S10D28** | 35.20±0.11 | 35.31±0.07 | 38.05±0.15 | 43.56±1.20 |
| **K1T24S10D20** | 36.98±0.03 | 39.03±0.32 | 37.26±0.04 | 36.84±0.15 |  | **K3T28S15D28** | 35.02±0.08 | 36.75±0.09 | 37.50±0.35 | 43.28±0.66 |
| **K1T24S15D20** | 35.80±0.05 | 37.96±0.35 | 35.98±0.15 | 36.63±0.24 |  | **K3T32S10D28** | 35.19±0.07 | 35.43±0.12 | 37.91±0.39 | 42.38±1.15 |
| **K1T28S10D20** | 36.02±0.10 | 36.89±0.62 | 36.07±0.71 | 35.83±0.53 |  | **K3T32S15D28** | 35.08±0.45 | 35.86±0.10 | 37.78±0.32 | 38.57±0.07 |
| **K1T28S15D20** | 37.01±0.31 | 37.10±0.21 | 36.64±0.64 | 35.96±0.12 |  | **K4T24S10D28** | 35.95±0.08 | 35.74±0.05 | 37.06±0.12 | 42.27±0.48 |
| **K1T32S10D20** | 35.91±0.10 | 37.99±0.37 | 36.37±0.01 | 36.29±0.28 |  | **K4T24S15D28** | 36.63±0.29 | 37.22±0.62 | 35.86±0.04 | 42.41±0.29 |
| **K1T32S15D20** | 36.02±0.13 | 37.29±0.11 | 36.67±0.13 | 36.13±0.14 |  | **K4T28S10D28** | 35.76±0.10 | 35.75±0.06 | 35.95±0.23 | 43.66±0.44 |
| **K2T24S10D20** | 36.73±0.14 | 35.72±0.28 | 36.49±0.10 | 36.37±0.52 |  | **K4T28S15D28** | 35.49±0.18 | 35.59±0.24 | 35.70±0.18 | 44.40±1.35 |
| **K2T24S15D20** | 35.35±0.11 | 35.44±0.24 | 35.58±0.11 | 36.10±0.10 |  | **K4T32S10D28** | 35.60±0.05 | 35.49±0.11 | 35.75±0.05 | 48.62±3.56 |
| **K2T28S10D20** | 35.64±0.05 | 34.71±0.18 | 35.81±0.06 | 35.69±0.07 |  | **K4T32S15D28** | 36.02±0.22 | 35.79±0.09 | 35.81±0.04 | 58.50±3.23 |
| **K2T28S15D20** | 36.02±0.27 | 35.03±0.09 | 36.15±0.21 | 35.81±0.23 |  | **K5T24S10D28** | 36.37±0.04 | 38.36±0.39 | 38.9±0.09 | 41.43±0.32 |
| **K2T32S10D20** | 35.49±0.21 | 34.90±0.11 | 35.79±0.23 | 35.84±0.06 |  | **K5T24S15D28** | 36.90±0.05 | 39.33±1.29 | 39.06±0.12 | 43.84±2.40 |
| **K2T32S15D20** | 35.61±0.02 | 35.52±0.09 | 36.21±0.23 | 35.82±0.02 |  | **K5T28S10D28** | 36.14±0.12 | 37.78±0.23 | 39.13±0.17 | 41.84±0.51 |
| **K3T24S10D20** | 36.29±0.15 | 38.96±0.34 | 36.32±0.15 | 36.34±0.14 |  | **K5T28S15D28** | 36.48±0.39 | 38.51±0.17 | 38.96±0.21 | 41.52±0.28 |
| **K3T24S15D20** | 36.64±0.33 | 37.99±0.20 | 37.25±0.25 | 36.86±0.04 |  | **K5T32S10D28** | 35.87±0.16 | 38.41±0.56 | 38.59±0.08 | 56.91±1.59 |
| **K3T28S10D20** | 35.20±0.11 | 37.67±0.05 | 35.50±0.04 | 35.45±0.15 |  | **K5T32S15D28** | 36.17±0.06 | 37.89±0.13 | 38.43±0.15 | 58.34±2.64 |
