# Supplementary material for: Spectral Pattern of Chocolate Production: Early Detection of Quality Problems
Source: J Food Sci. 2026 Jul 20;91(7):e71269. doi: 10.1111/1750-3841.71269 (PMC13383598; doi:10.1111/1750-3841.71269)
Supplement: Supplementary file 1 — Table S1. Hardness changes of chocolate samples within time. Table S2. Water activity changes of chocolate samples. Table S3. Color changes of chocolate samples within time. Table S4. WI changes of chocolate samples within time. Table S5. Moisture, water activity, and particle size values of variously conched samples after conching process. [file JFDS-91-0-s001.zip › jfds71269-sup-0001-TableS5.docx]

| **Sample code** | **Moisture (%)** | **Water activity (a_w_)** | **Particle size (D50)** |
| --- | --- | --- | --- |
| **K1** | 1,02±0,06^ab^ | 0,30±0,02^b^ | 14,70±1,25^bc^ |
| **K2** | 0,99±0,06^b^ | 0,34±0,02^ab^ | 30,82±1,74^a^ |
| **K3** | 0,72±0,04^c^ | 0,37±0,02^a^ | 11,27±0,21^c^ |
| **K4** | 1,23±0,07^a^ | 0,29±0,03^b^ | 17,17±1,36^b^ |
| **K5** | 0,92±0,05^bc^ | 0,31±0,03^ab^ | 31,75±2,76^a^ |

*According to Tukey test, different letters (a,b and c) in each column mean a significant difference (p < 0.05)
